# The beneficial effect of fluoxetine on behavioral and cognitive changes in chronic experimental Chagas disease unveils the role of serotonin fueling astrocyte infection by *Trypanosoma cruzi*

**DOI:** 10.1371/journal.pntd.0012199

**Published:** 2024-05-22

**Authors:** Glaucia Vilar-Pereira, Daniel Gibaldi, Leda Castaño-Barrios, Andrea Alice da Silva, Isabela Resende Pereira, Otacílio Cruz Moreira, Constança Britto, Hílton Antônio Mata dos Santos, Raquel de Oliveira Lopes, Luzineide Wanderley Tinoco, Wilson Oliveira, Joseli Lannes-Vieira

**Affiliations:** 1 Laboratório de Biologia das Interações, Instituto Oswaldo Cruz/Fiocruz, Rio de Janeiro, Brazil; 2 Laboratório Multidisciplinar de Apoio à Pesquisa em Nefrologia e Ciências Médicas, Departamento de Patologia, Faculdade de Medicina, Universidade Federal Fluminense, Niterói, Brazil; 3 Laboratório de Virologia e Parasitologia Molecular, IOC/Fiocruz, Rio de Janeiro, Brazil; 4 Laboratório de Biologia Molecular e Doenças Endêmicas, IOC/Fiocruz, Rio de Janeiro, Brazil; 5 Escola de Farmácia, Universidade Federal do Rio de Janeiro, Rio de Janeiro, Brazil; 6 Laboratório de Análise e Desenvolvimento de Inibidores Enzimáticos, Universidade Federal do Rio de Janeiro, Rio de Janeiro, Brazil; 7 Programa de Pós-Graduação em Farmacologia e Química Medicinal, Instituto de Ciências Biomédicas, Universidade Federal do Rio de Janeiro, Rio de Janeiro, Brazil; 8 Laboratório Multiusuário de Análises por Ressonância Magnética Nuclear (LAMAR), Instituto de Pesquisas de Produtos Naturais (IPPN), Universidade Federal do Rio de Janeiro, Rio de Janeiro, Brazil; 9 Ambulatório de Doença de Chagas e Insuficiência Cardíaca, Pronto Socorro Cardiológico de Pernambuco (PROCAPE)/Universidade de Pernambuco, Recife, Brazil; US Food and Drug Administration, UNITED STATES

## Abstract

**Background:**

In Chagas disease (CD), a neglected tropical disease caused by the parasite *Trypanosoma cruzi*, the development of mental disorders such as anxiety, depression, and memory loss may be underpinned by social, psychological, and biological stressors. Here, we investigated biological factors underlying behavioral changes in a preclinical model of CD.

**Methodology/Principal Findings:**

In *T*. *cruzi*-infected C57BL/6 mice, a kinetic study (5 to 150 days postinfection, dpi) using standardized methods revealed a sequential onset of behavioral changes: reduced innate compulsive behavior, followed by anxiety and depressive-like behavior, ending with progressive memory impairments. Hence, *T*. *cruzi*-infected mice were treated (120 to 150 dpi) with 10 mg/Kg/day of the selective serotonin reuptake inhibitor fluoxetine (Fx), an antidepressant that favors neuroplasticity. Fx therapy reversed the innate compulsive behavior loss, anxiety, and depressive-like behavior while preventing or reversing memory deficits. Biochemical, histological, and parasitological analyses of the brain tissue showed increased levels of the neurotransmitters GABA/glutamate and lipid peroxidation products and decreased expression of brain-derived neurotrophic factor in the absence of neuroinflammation at 150 dpi. Fx therapy ameliorated the neurochemical changes and reduced parasite load in the brain tissue. Next, using the human U-87 MG astroglioma cell line, we found no direct effect of Fx on parasite load. Crucially, serotonin/5-HT (Ser/5-HT) promoted parasite uptake, an effect increased by prior stimulation with IFNγ and TNF but abrogated by Fx. Also, Fx blocked the cytokine-driven Ser/5-HT-promoted increase of nitric oxide and glutamate levels in infected cells.

**Conclusion/Significance:**

We bring the first evidence of a sequential onset of behavioral changes in *T*. *cruzi*-infected mice. Fx therapy improves behavioral and biological changes and parasite control in the brain tissue. Moreover, in the central nervous system, cytokine-driven Ser/5-HT consumption may favor parasite persistence, disrupting neurotransmitter balance and promoting a neurotoxic environment likely contributing to behavioral and cognitive disorders.

## Author summary

In chronic Chagas disease, illness caused by *Trypanosoma cruzi* infection, anxiety, depression, and memory decline are mostly related to social and psychological stressors. Here, we described the sequential onset of behavioral and cognitive changes in *T*. *cruzi*-infected mice, supporting a role for biological stressors. Indeed, in chronically infected mice, the behavioral changes parallel increased levels of the neurotransmitters GABA/glutamate and lipid peroxidation products and decreased expression of the neurotrophin BDNF. Therapy with the selective serotonin reuptake inhibitor and antidepressant fluoxetine ameliorated, prevented, or reversed behavioral and cognitive changes, improved neurochemical alterations, and, surprisingly, reduced parasite load in the brain. *In vitro*, serotonin fueled parasite uptake by U-87 MG cells, a human astrocyte-like cell lineage. Serotonin effects were increased by prior interferon-gamma (IFNγ) and tumor necrosis factor (TNF) stimulation but abrogated by fluoxetine and related to nitric oxide and glutamate levels in culture supernatants. Thus, the cytokine-driven consumption of serotonin may fuel *T*. *cruzi* uptake by astrocytes, sustaining brain parasitism, reducing serotonin availability, dysregulating the neurotransmitter and neurotrophin networks, and favoring a neurotoxic milieu, which may contribute to behavioral and cognitive disorders. Therefore, fluoxetine therapy, which interferes with these biological processes, may improve the quality of life of Chagas disease patients.

## Introduction

In the last two decades, mental illnesses such as mood disorders, anxiety, depression, and memory impairments have gained much attention in patients with pathogen-triggered chronic diseases caused by hepatitis viruses [1], enteroviruses [2], human immunodeficiency virus [3], SARS-COV2 [4]), bacteria [5], and protozoans, such as toxoplasmosis [6] and malaria [7]. Nonetheless, the biological and molecular factors underpinning behavioral and cognitive changes in infectious diseases remain unclear.

Mental disorders also affect patients with Chagas disease (CD), a neglected tropical disease caused by the protozoan parasite *Trypanosoma cruzi*, which mainly afflicts people born and living in poor conditions in Latin America [8]. However, due to human migration, CD has been globalized with increased prevalence and potential to become a health problem in non-endemic regions of the United States of America, Asia, Oceania, and Europe [9]. In recent decades, much attention has been paid to the *T*. *cruzi* parasite-host cell interactions, immunological traits, pathological aspects, and clinical presentation of CD. These studies mainly focus on the cardiac form of CD, which afflicts approximately 30% of the patients 10–30 years after infection [10]. In chronic CD patients, a neurological form is associated with co-infections, immunosuppression, and neurobehavioral changes as sequelae of strokes [11]. Despite the importance of mental disorders and their possible impact on patient’s quality of life and clinical management, few clinical and preclinical studies have explored behavioral and cognitive aspects of CD. Anxiety disorders, mood changes, and depressive episodes have been described in CD patients [12]. Further, a study using the Beck Depression Inventory showed that only 42% of the patients with CD consider having a positive quality of life [13]. Moreover, 37 to 40% of the CD patients met diagnostic criteria for a current depressive disorder, regardless of the presence of chronic Chagas’ heart disease or preserved cardiac functions [12–15]. In chronic CD patients, cognitive dysfunction has been supported by a deficiency in orientation, attention, non-verbal reasoning, information processing, learning, and memory deficits [13,16–19]. Experimental models have reproduced some aspects of mental disorders described in CD patients. Acute and chronically *T*. *cruzi*-infected rats show sleep and memory impairments [20]. Single-point studies revealed that chronically *T*. *cruzi*-infected mice show signs of anxiety, depressive-like behavior [21], and memory deficit [22], independently of sickness behavior [23]. Moreover, in infected mice, depressive behavior has been associated with high serum levels of tumor necrosis factor (TNF) [21], and memory loss has been related to parasite persistence and oxidative stress in the central nervous system (CNS) [22]. Thus, independent of the involvement of social and psychological stressors as potential determinants of mental disorders in CD patients, mostly afflicted by stigmatization, discrimination, and poverty [8], preclinical studies support that biological stressors may contribute to behavioral changes in CD.

Anxiety and depressive disorders may act as comorbidity factors for memory decline, which may be associated with mental distress and the onset of depressive symptoms. Further, mental disorders may have a complex multifactorial nature and a diversity of triggers and biological stressors, some of them familiar to neurodegenerative and aging-related cognitive diseases, such as neuroinflammation [24], oxidative stress [25] and disturbs of the networks of neurotrophins and neuromediators. In this context, the neurotrophin brain-derived neurotrophic factor (BDNF) plays a key role in neuronal survival, growth, and plasticity, crucial processes for learning and memory formation [26]. Further, the gamma-aminobutyric acid (GABA) and glutamate are, respectively, inhibitory and excitatory neurotransmitters, and high concentrations of these molecules can disrupt brain physiology, contributing to behavioral disorders such as depression [27]. Serotonin (Ser/5-HT), a metabolite of the tryptophan pathway, is another important neurotransmitter that regulates practically all behavioral processes, including depression and cognitive functions. A cytokine-driven imbalance of the tryptophan pathway involving the tryptophan-catabolizing enzyme indoleamine 2,3-dioxygenase (IDO) favors the production of kynurenine and quinolinic acid, decreasing the availability of Ser/5-HT, and contributing to neuronal death and behavioral changes [28,29]. Thus, here we hypothesized that *T*. *cruzi* infection may provide biological stressors, disrupting the networks of neurotrophins and neuromediators, and opening the doors for behavioral and cognitive changes in the chronic phase of CD.

First, we conducted a study to establish an experimental model to challenge the sequential onset of behavioral and cognitive changes in *T*. *cruzi*-infected mice. With these data, we tested the effects of the selective serotonin reuptake inhibitor (SSRI) fluoxetine (Fx), an antidepressant that also favors neuroplasticity and cognition [29], on clinical alterations and canonical biological stressors such as alterations of GABA and glutamate, increase in lipid peroxidation as a marker of oxidative stress, and a decrease in the expression of the neurotrophin BDNF. Lastly, the *in vivo* findings led us to use an *in vitro* model of human astrocytes to investigate the participation of the neurotransmitter serotonin (5-hydroxytryptamine; Ser/5-HT) and cytokine-driven processes in crucial biological traits, raising the discussion that they may contribute to parasite persistence in the CNS and behavioral and cognitive alterations.

## Materials and methods

### Ethics statement

All experimental procedures were conducted under the recommendations of the Guide for the Care and Use of Laboratory Animals of the Brazilian National Council of Animal Experimentation (https://www.mctic.gov.br/mctic/opencms/institucional/concea/) and the Brazilian Federal Law 11.794 (October 8^th^, 2008). The procedures were approved by the Ethical Commission on Animal Use of Fiocruz and Oswaldo Cruz Institute/Fiocruz (licenses LW10/14 and L006/2018). The *in vivo* data were obtained from three independent experiments (Register Books #47, #53, and #73, LBI/IOC-Fiocruz).

### Experimental design

The experimental protocol for the *in vivo* studies is described in Workflow (S1 Fig). The Institute of Science and Technology in Biomodels (ICTB) of the Oswaldo Cruz Foundation (Fiocruz) provided 122 C57BL/6 strain (H-2^b^) 5-7-week-old female mice. After arrival, mice were housed in polypropylene cages with Pinus sawdust and randomly grouped into 3–5 mice per cage. The cages were kept in microisolators, and mice received water and grain-based chaw food *ad libitum*. Mice were adapted for 10–14 days in a plastic igloo-enriched cage in specific pathogen-free conditions, with light and noise control to minimize stress. After this period, mice were infected and analyzed according to the experimental protocols (S1 Fig), using time points previously described, thus characterizing the onset of the acute and chronic phases in this experimental model [30]. Groups were composed as follows: **Kinetic study** (total 72 mice; 12 mice/time point: 5 NI and 7 *T*. *cruzi*-infected mice), when mice were evaluated at different time points during the acute phase (5–90 dpi) and chronic phase (118–150 dpi): 5–9 dpi; 33–39 dpi; 54–58 dpi; 82–90 dpi; 118–120 dpi–referred to as “120 dpi” (also referred to as pre-therapy–group 1); and 149–152 dpi–referred to as “150 dpi”. **Pre-therapy** (group 2): non-infected (NI) controls (5 mice); *T*. *cruzi*-infected (7 mice); analyzed at 120 dpi. **Fx-therapy experiments** (therapy 120–151 dpi; analysis at 149–152 dpi–referred to as “150 dpi”; total 38 mice: 2 repetitions with 19 mice): non-infected (NI) controls (5 mice); vehicle-treated *T*. *cruzi*-infected (7 mice); Fx-treated *T*. *cruzi*-infected (7 mice). At arrival, mice were grouped, cages numbered and randomly sorted for the experimental infection, and analysis at the indicated time points.

### Infection by *Trypanosoma cruzi* and clinical follow-up

Mice were infected intraperitoneally with 100 blood trypomastigote (bt) forms of the Discrete Typing Unit Type I Colombian *T*. *cruzi* strain suspended in 0.2 mL of sterile saline buffer, and parasitemia was performed weekly, as previously described [30]. Weekly, death was recorded, and clinical signs were analyzed (piloerection, apathy, prostration, mobility, posture, aggressive behavior, pain). Body weight loss (which may reveal loss of appetite, diarrhea, and absorption disturbance) was assessed using a rodent weighing scale (ED623S-OCE, Sartorius scale, USA). Exclusion criteria were negative parasitemia (35 to 45 dpi), any death outside of planned euthanasia and according to pre-established humane endpoints conditions such as body weight loss (≥ 30% of the initial weight), injuries from fights, pain, posture, ataxia, and immobility.

### Fluoxetine treatment

Animals were treated daily, from 120 to 151 dpi, by gavage with 0.1 mL of the vehicle (Veh) apyrogenic vaccine-graded water (BioManguinhos, Fiocruz, Brazil) or 0.1 mL of Veh containing 10 mg/Kg/day of Fx (Prozac, Eli Lilly, Brazil), previously used in acutely infected mice [21].

### Behavioral tests

Behavioral tests were conducted between 8:00 am and 3:00 pm and recorded using a DSC-DVD810 video camera (Sony, USA). These tests were conducted in a controlled environment with 12 hours of light and 12 hours of the dark cycle at a stable room temperature of 22 ± 2°C and a noise level of approximately 40 dB produced by an air conditioner. This setting was designed to reduce stress and enhance the animals’ familiarity with the environment. Different groups of animals were submitted to behavioral tests, including 5–150 dpi (Kinetic study), 120 dpi (Pre-therapy), and 149–151 dpi (Therapy experiments). The mice were not retested, but all were reused in different tests to reduce the number of animals used. Behavioral tests were performed from less stressful to the more stressful: open field test (exploratory activities—OFT1; spatial habituation memory test—OFT2), novel object recognition memory test (NORT), elevated plus maze test (EPMT), marble burying test (MBT), tail suspension test (TST), grip strength meter test (GSMT), previously settled [22,23].

Mice tend to explore new environments, which is the basis of the open field test (OFT). On the first day (OFT1), mice are placed in the upper corner of the testing area and allowed to explore for 5 minutes [23]. The OFT1 allows the analysis of mice’s exploratory/locomotor activity (total crossed lines) and anxiety (central crossed lines). The number of crossed lines was registered, and the following parameters were analyzed: (i) total locomotor activity, i.e., when the animals crossed each grid line with all four paws in the total area; (ii) inner locomotor activity, i.e., when the animals crossed each grid line with all four paws in the central area. This first day is also considered the training session (day 1) to evaluate the spatial habituation memory [22]. The long-term memory test was performed 24 hours after the training (day 2), in which the procedure was repeated, and the 5-minute session was recorded. Memory retention was evaluated by counting the total lines crossed during the test session [31]. The individual baseline differences were corrected using the change ratio score to compare behavior during the initial (day 1) and final session (day 2), and data are shown as discrimination index, as follows: number of crossed lines day 2 / (number of crossed lines day 1 + number of crossed lines day 2), as described [32]. The NORT is based on the innate preference of NI normal rodents to explore a novel object rather than a familiar one [33]. The test was initiated 2 hours after the OFT2 [22]. For NORT, the discrimination index was calculated as follows: time exploring the novel object / (time exploring the novel object + time exploring the familiar object), as previously shown [34].

Anxiety-like behavior was also assessed using EPMT, based on the natural tendency of mice to explore a new environment [23]. The behavioral parameters related to anxiety were the (i) number of entries and (ii) time spent in the open arms. An arm entry was scored when all four paws of the animal were placed on an arm. Mice exhibit a repetitive compulsory burying behavior in the presence of aversive stimuli; this is based on the MBT. Here, glass beads were used as aversive stimuli, and the number of buried glass beads in sawdust was registered at 5, 10, 15, 20, 25, and 30 minutes [23].

The immobile posture or apathetic behavior are interpreted as depressive-like behavior in TST and FST, short-term moderately stressful or uncomfortable inescapable conditions [35]. Here, we used the tail suspension apparatus (TST, Insight, Brazil), and the test was performed as previously described [36]. Data are expressed as immobility time in seconds (s). The FST assay was performed as previously described [23]. After the first 2 minutes of habituation, the total duration of immobility was measured over 4 minutes of the test. The mouse was immobile when it remained floating passively in the water or was making slight movements to keep its head above the water. The animal was dried with gauze, warmed in the bioheater (EFF 307, Insight, Brazil) for 2 minutes, and returned to its cage. Data are expressed as immobility time in seconds (s). Muscle strength was assessed using the grip strength meter (EFF 305, Insight, Brazil), according to the manufacturer’s instructions, as previously published [23]. Data are presented as the mean of strength intensity = gram-force (gf)/body weight (g).

### Electrocardiogram registers (ECG)

Mice were tranquilized intraperitoneally with diazepam (10 mg/Kg). Data were acquired, and we measured heart rate (beats per minute [bpm]), PR, and QT intervals in milliseconds. Calculation was performed to obtain physiologically relevant values for the heart rate-corrected QT interval (QTc), as previously shown [37].

### Blood obtention, euthanasia and encephalon tissue obtention and histology

After topical eye drops anesthesia, mice were bled by the orbital plexus, and serum was stored at -80°C. Mice were euthanized at the endpoints (acute phase: 35–40 dpi; and chronic phase: 120 dpi and 150 dpi) using CO_2_ inhalation in an appropriate chamber, allowing 70% of CO_2_ saturation for 2–3 minutes, followed by decapitation. Encephala and hearts were collected and weighed. Considering the importance of the cerebral cortex and hippocampus for behavioral and cognitive changes, these areas were dissected [38]. After addition of RNA later (Thermo Fisher Scientific, USA), the tissues were stored at -80°C for the following assays: TNF, BDNF, IDO and SERT/5-HTT gene expression; lipid peroxidation by evaluation of tissue levels of thiobarbituric acid reactive species (TBARS); GABA/glutamine determination by nuclear magnetic resonance; and parasite load determination by quantitative PCR (qPCR).

The encephalon was removed for histological studies, fixed in buffered formalin 10%, dehydrated, and embedded in paraffin. Five μm-thick sections were prepared and stained with hematoxylin and eosin, and two sections per encephalon tissue were examined using light microscopy. Representative images were digitized using a Sight DS-U3 color-view digital camera adapted to an Eclipse Ci-S microscope and analyzed with the digital morphometric apparatus NIS Elements BR version 4.3 software (Nikon Corporation, Sendai, Japan).

### Determination of parasite load in the CNS by quantitative PCR (qPCR)

Mice were euthanized, and the encephalon was removed and dissected, as described above. DNA was extracted from dissected cerebral cortex and hippocampus samples, and parasite load was determined and quantified using a standard curve, as previously described [22]. The parasite load was normalized by the tissue mg equivalents by dividing the *T*. *cruzi* quantity by the mass of mice tissue equivalents (cortex or hippocampus) and expressed as “parasite equivalents per mg of tissue” [22].

### Real-time quantitative RT-PCR for TNF, IFNγ, SERT, IDO and BDNF

For real-time quantitative RT-PCR (RT-qPCR), total RNA from hearts and encephala samples was extracted using RNeasy Mini kit-Qiagen (74104, Qiagen, USA) and TRI Reagent (Sigma-Aldrich, USA), respectively. After quantification and treatment with DNAse I, reverse transcriptase reactions were performed on 5 μg RNA using the Superscript III kit (18080–051, Invitrogen, USA) according to the manufacturer’s instructions. All RNA samples were reverse transcribed simultaneously to minimize the interassay variation associated with the reverse transcription reaction, and reverse transcriptase negative control was used in each reverse transcription to monitor the absence of DNA contamination. Real-time RT-PCR was performed on an ABI Prism 7500 Fast (Applied Biosystems) using TaqMan gene expression assays for the cytokines TNF (Mm00443258_m1) and IFNγ (Mm01168134_m1), serotonin transporter 1 (SERT/5-HTT; Mm00439391_m1) and IDO (Mm00492586_m1) purchased from Applied Biosystems (USA). Reactions were performed in duplicate according to the manufacturer’s instructions using a 2 μL cDNA template for each reaction in a total volume of 20 μL. The relative quantitative measurement of target gene levels was calculated using the Expression Suite software (Applied Biosystems) by the ΔΔCt method [39]. The glyceraldehyde 3-phosphate dehydrogenase (GAPDH; Mm99999915_g1) and the β actin (Mm00607939_s1) genes were used as endogenous housekeeping reference genes. The RT-qPCR products and a molecular weight marker were electrophoresed in 1% agarose gel and stained with Nancy-520 (Sigma, Switzerland). Results were expressed as a fold increase in comparison with the NI controls.

For BDNF studies, cerebral cortex and hippocampus tissues were removed, weighed, and stored in RNA later (AM7021, Thermo Fisher Scientific, USA) in a freezer at -80°C. Total RNA was extracted from the same sample used to quantify the parasite load using TRI-Reagent (Sigma-Aldrich, St. Louis, MO, USA). After extraction, the RNA was purified using the RNeasy kit (QIAGEN, USA) according to the manufacturer’s instructions. After quantification and treatment with DNAse I, cDNA was synthesized with the Superscript VILO MasterMix (Thermo Fisher Scientific, USA). The total reaction (20 μL) for the reverse transcription contained 4 μL of SuperScript VILO MasterMix, 4 μL of H_2_O DEPC, and 2.5 μg of total RNA. The mixture was incubated at 25°C for 10 minutes, at 42°C for 60 minutes for transcription, and 85°C for 5 minutes for termination of the reaction. Reverse transcriptase negative control was used in each reverse transcription to monitor the absence of DNA contamination. The cDNA dosage was performed on the Qubit 3.0 Fluorometer using the ssDNA kit (Thermo Fisher Scientific, USA). For gene expression analysis, the RT-qPCR reaction was performed using the following TaqMan Assays (Applied Biosystems, USA), according to the manufacturer’s instructions: BDNF neurotrophic factor (Mm04230607_s1) as the target gene and GAPDH (glyceraldehyde-3-phosphate dehydrogenase; Mm99999915_g1) and HPRT (hypoxanthine-guanine phosphoribosyltransferase; Mm01545399_m1) as reference genes. The reaction mixture (10 μL) contained 2 μL of cDNA from each sample, 5 μL of Master Mix TaqMan 2x, 0.5 μL of the above-described probes and 2.5 μL of H_2_O DEPC. All reactions were performed in duplicate. PCR cycle conditions were as follows: 95°C for 10 minutes for initial denaturation, 40 cycles of 95°C for 15 sec for denaturation, and 60°C for 1 minute for annealing/extension (Viia7, Applied Biosystems, USA).

### Determination of glutamate and GABA neurotransmitters in the CNS by nuclear magnetic resonance

The cortex and hippocampus were analyzed for GABA and glutamate neurotransmitters using nuclear magnetic resonance. The dissected cortex and hippocampus were collected and deeply frozen. Samples were dissolved in 99.9% D_2_O (Cambridge Isotope Laboratories, Inc., USA) with 0.5 mM 98% 3-(Trimethylsilyl) propionic-2,2,3,3-*d*_4_ acid sodium salt (Cambridge Isotope Laboratories, Inc., USA), used as a reference for the chemical shift (0.00 ppm) and standard for quantitative analysis. ^1^H NMR spectra were acquired at 25°C on a VNMRS-500 (Agilent, USA) spectrometer at 499.78 MHz. Spectra were acquired with a spectral width of 7022 Hz, with 16384 points and 64 accumulations. A relaxation interval (d1) of 35 s was used for quantitative analysis. Nuclear magnetic resonance spectra were processed with the MestReNova v10.0.1–14719 program.

### Lipid peroxidation evaluation in the CNS

Mice were euthanized, the encephalon removed, the cortex and hippocampus dissected, and homogenates prepared. Malondialdehyde and other TBARS, products of degradation of hydroperoxides and lipid peroxides formed during the oxidation of fatty acids, were determined, as previously described [22]. Ten percent (w/v) of tissue homogenate was mixed with 8.1% sodium dodecyl sulfate, 20% acetic acid pH 3.5, and 0.8% TBA and incubated at 95°C for 1 hour. After incubation, the reaction product was extracted with n-butanol (1:1) and read using a spectrophotometer (Spectra Max M5, Molecular Devices, USA) at 532 nm.

### Determination of cytokines and BDNF in sera

The BD Cytometric Bead Array (CBA) Mouse Inflammation kit (552364, BD Bioscience) measured serum cytokine levels using standard calibration curves according to the manufacturer’s recommendations. The samples were analyzed using the 13-Color CytoFLEX-S flow cytometer (BeckmanCoulter, USA). Cytokine concentrations were determined and expressed in pg/ mL using the FCAP Array Software. BDNF in sera was assessed with the Biosensis BDNF *Rapid* ELISA Kit (BEK-2211-2P, Australia), using a standard curve of 250 to 7.8 pg/mL with a limit of detection down to 2 pg/mL BDNF and read using a spectrophotometer (Spectra Max M5, Molecular Devices, USA) at 450 nm.

### *In vitro* assays using the human U-87 MG cell lineage

The glioblastoma cell line U-87 MG, a Ser/5H-T-responsive cell [40], was purchased from Banco de Células do Rio de Janeiro (BCRJ, Rio de Janeiro, RJ, Brazil). Cells were cultivated in Dulbecco’s modified Eagle medium (DMEM) supplemented with 10% fetal bovine serum (FBS) and 1% penicillin-streptomycin (Sigma-Aldrich, St. Louis, MO, USA) and maintained at 37°C in a 95% humid atmosphere with 5% CO_2_. Cell culture dissociation was performed by replacing the medium with 0.25% trypsin/0.02% EDTA in PBS. Cells were plated at a density of 10^5^ cells per 13-mm diameter poly-ornithine-coated coverslips in 24-well plates (NUNC, Roskilde, Denmark).

Trypomastigote forms of the Colombian *T*. *cruzi* strain were obtained from cultures of the Vero cell line (CCL81 ATCC, Manassas, VA, USA), as previously described [41]. We used a multiplicity of infection (MOI) of 5:1 (parasite: cell) and interaction periods of 4 hours at 37°C, 95% humidity, and 5% CO_2_. According to the experiments, supernatants were collected and stored at -20°C. Coverslips were washed twice with warm PBS, and the cells were fixed in fresh 4% paraformaldehyde for 10 minutes. After three washes with PBS, the coverslips were mounted with prolonged gold antifade reagent with 4’,6-diamidino-2-phenylindole (DAPI; Life Technologies, USA) and examined and photo documented using a fluorescence microscope (Leica DFC300FX, Germany). The numbers of NI and *T*. *cruzi*-infected astrocytes (500 cells/coverslip) and intracellular forms per infected cell were counted, and we determined the Infection Index by multiplying the percentage of infected cells by the number of parasites per infected cell. The figure legends provide each experiment’s details.

To explore the effect of Ser/5-HT (Serotonin hydrochloride, 5-HT, 5-hydroxytryptamine, 14332, Cayman Chemicals, USA) and Fx (Fluoxetine hydrochloride, LY110140, 14418, Cayman Chemicals, USA) on U-87 MG, initially solutions at a final concentration of 1 to 1000 μM diluted in the maximum 1% dimethyl sulfoxide (DMSO; Sigma Aldrich, St. Louis, MO, USA) in DMEM-10% FBS were added to astrocytes to test cytotoxicity. Cell viability was observed by the reduction of MTT (3–4,5-dimethylthiazol-2-yl-2,5-diphenyltretrazolium bromide; Sigma-Aldrich, Steinheim, Germany). After 5 minutes, viable cells convert MTT into insoluble purple formazan, which was solubilized with DMSO, and the optical density was evaluated at 490 nm using a microplate reader (Asys Expert Plus, Biochrom, Cambridge, UK). After a test of concentrations, Ser/5-HT was used at 1 or 10 μM 1 hour before infection, as described in the experimental design and indicated in corresponding figure legends. In *in vitro* assays, Fx was added at 20 μM [42] 15 minutes before adding Ser/5-HT to cell cultures.

To investigate the effects of IFNγ and TNF on *T*. *cruzi* infection, U-87 MG cells were plated (10^5^ cells per 13 mm poly-ornithine-coated coverslip in a 24-well plate), allowed to adhere for 24 hours, washed three times with warm PBS, and added fresh DMEM-10% FBS. Cultures were left untreated or were pre-treated with recombinant human IFNγ (rIFNγ, 10 ng/mL, eBioscience, San Diego, CA, USA) or TNF (rTNF, 10 ng/mL, eBioscience, San Diego, CA, USA), according to the experimental designs as indicated in figure legends. Cell cultures were treated with the anti-TNF chimeric monoclonal antibody infliximab (10 μg/mL, Remicade, a gift of Schering-Plough, Brazil) to evaluate the role of rIFNγ-induced TNF in astrocyte infection, 30 minutes before the addition of murine or human rIFNγ. Nitric oxide (NO) concentrations in cell culture supernatants were assessed by Griess reaction, the optical density of the product was evaluated at 540 nm using a microplate reader (Asys Expert Plus, Biochrom, Cambridge, UK), and sample concentrations were estimated based on a standard curve (0.8–100 μM sodium nitrite). We used a commercial kit to detect glutamate in cell culture supernatants (MAK004, Glutamate Assay Kit, Sigma-Aldrich, St. Louis, MO, USA), according to manufacturer’s instructions. Sample concentrations were estimated using a standard curve (0.1–10 nM glutamate).

### Statistical analysis

The sample size was determined based on our group’s experience and previous studies using the model of experimental chronic chagasic cardiomyopathy [30,37] and behavioral alterations [21,22]; therefore, no formal sample size was calculated. The Shapiro-Wilk test was used to assess the data’s normality. For normally distributed data formed by two groups, differences were analyzed with the Student’s t-test. For normally distributed data of more than two groups, the difference between groups was analyzed using the parametric one-way ANOVA test, corrected with Turkey post hoc test with multiple comparisons, with a 95% confidence. Non-normally distributed data were analyzed using the non-parametric Kruskal-Wallis H test for groups with two groups, or one-way ANOVA on ranks for analysis with more than two groups followed by the post hoc Dunn’s multiple comparisons tests. Data from two or three independent experiments were grouped. All statistical tests were performed with GraphPad Prism 8.0.1 (La Jolla, CA, USA). Differences were considered statistically significant when *p <* 0.05.

## Results

### Sequential onset and progression of behavioral and cognitive changes in *T*. *cruzi*-infected mice

**Fig 1:**
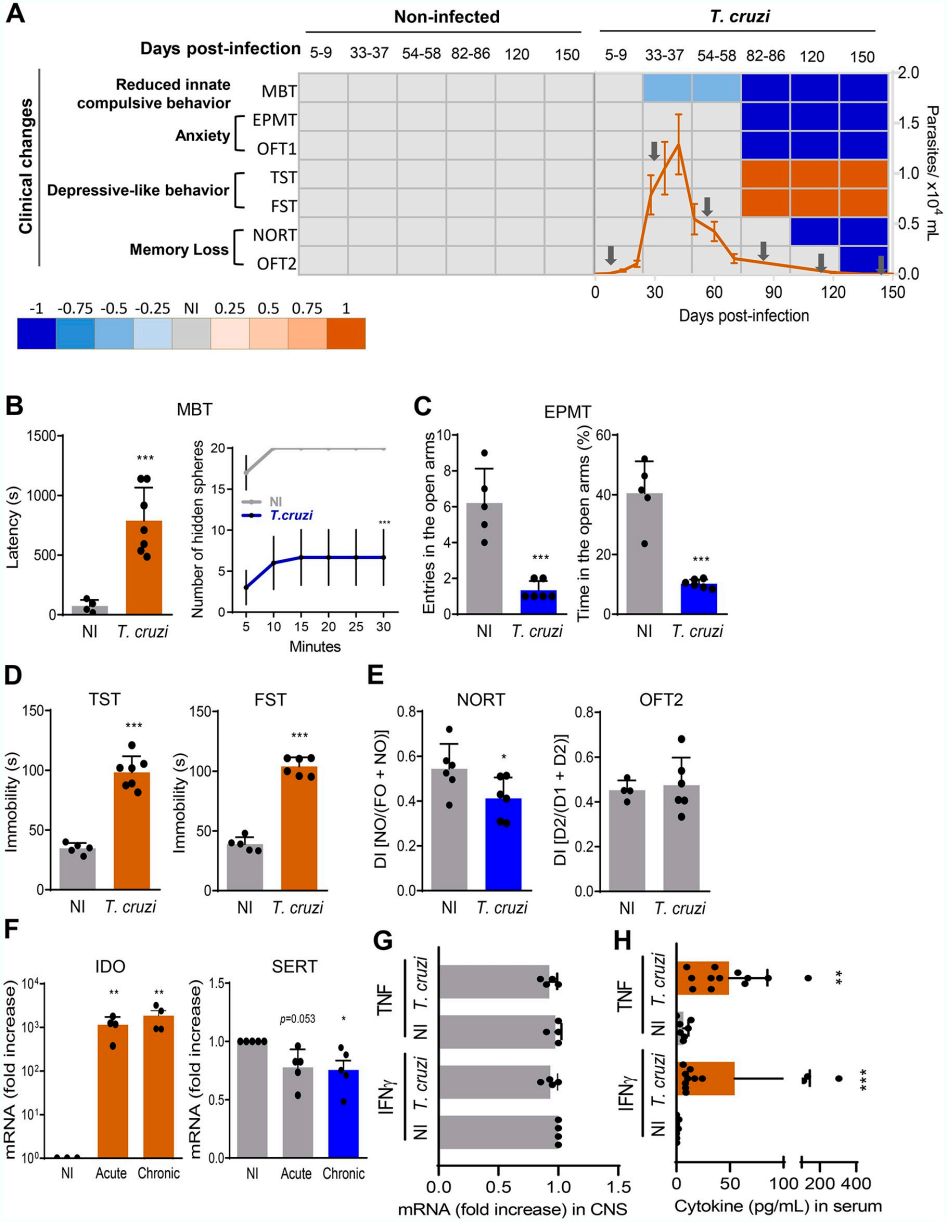
Behavioral, cognitive, and biological alterations in *Trypanosoma cruzi*-infected mice. (**A**) Heatmap corresponding to behavioral and cognitive traits in non-infected control mice (NI) and infected with 100 bt forms of the Colombian Type I strain (*T*. *cruzi*). The transverse axis of the heatmap represents the standardized tests applied to assess innate compulsive behavior (MBT), anxiety-like behavior (EPMT, OFT1), depressive-like behavior (TST, FST), and memory (NORT, OFT2). The longitudinal axis represents days postinfection (dpi) analyzed (from 5–9 up to 150 dpi). Each lattice color represents an analyzed parameter, with grey representing normal behavioral conditions, shades of blue representing degrees of decrease of an analyzed parameter, and shades of brown representing degrees of increase of a studied parameter. The results of the analyzed parameters denoted behavioral or neurocognition clinical changes. The parasitemia graph was overlaid (means ± SD), and arrows indicate the time points of clinical evaluation. (**B-E**) Behavioral and cognitive traits, at 120 dpi (pre-therapy time point): (**B**) Latency and the number of hidden spheres in 30-minutes marble burying test (MBT). (**C**) Number of entries and time in the open arms in an elevated plus maze test (EPMT). (**D**) Time of immobility in the tail-suspension test (TST) and forced-swim test (FST). (**E**) Discrimination index (DI) in the novel object recognition memory task (NORT) and the two-day exposition to the open field test (OFT2). (**F**) Expression of indoleamine 2,3-dioxygenase (IDO) and serotonin transporter 1 (SERT/5-HTT) in the encephalon tissue of acute (35–40 dpi) and chronically (120 dpi) *T*. *cruzi*-infected mice. (**G**) Expression of IFNγ and TNF in the encephalon tissue. (**H**) Levels of IFNγ and TNF in serum. Results representative of two independent experiments. Color code: Grey bars indicate normal, blue bars indicate a decrease and brown bars show increased values compared with NI controls. Each dot represents a mouse. The data are shown as the means ± SD. *, *p* < 0.05, **, *p* < 0.01 and ***, *p* < 0.001, *T*. *cruzi*-infected compared with NI controls.

All *in vivo* experiments were performed according to the experimental Workflow (S1 Fig). S1 Table shows all data used to build graphs. The low-dose inoculum of trypomastigote forms allowed 100% survival at 150 dpi. Firstly, we established a kinetic study of the onset, progression, and severity of behavioral and cognitive alterations in *T*. *cruzi*-infected mice. To summarize the results obtained using different tests and draw a holistic picture of the studied behavioral traits, we settled on a score-based heatmap, considering arbitrarily -1.0 as the maximum decrease or 1.0 as the maximum increase of the evaluated parameters. Then, we scored the data of the noticed abnormalities in other experimental time points compared to the top scorer (-1.0 or 1.0). The score-based heatmap (Fig 1A) summarizes the data obtained comparing *T*. *cruzi*-infected mice (7 mice/time point) with sex- and age-matched NI controls (5 mice/point).

Mice show an innate compulsive behavior of burying aversive materials in their environment. As shown in the score-based heatmap (Fig 1A), the innate compulsive behavior assessed by the MBT is preserved at 5–9 dpi but partially lost at 33–37 dpi (S2A Fig) before the parasitemia peak observed at 42–45 dpi (Fig 1A). This behavioral abnormality progressed to a more severe form after parasitemia control (82–86 dpi; Figs 1A and S2A), a condition that persisted during the chronic phase of infection, achieving the maximum detected score when parasitemia is intermittent or not apparent at 120 and 150 dpi (Fig 1A). Anxiety-like behavior, expressed as a decrease in the exploration of open arms in EPMT and avoidance of the central area in OFT1, was detected to a similar degree in all analyzed *T*. *cruzi*-infected mice after 82 dpi and up to 150 dpi when parasitemia was controlled (Fig 1A). Increased immobility in TST and FST was noticed in *T*. *cruzi*-infected mice after 82 dpi and up to 150 dpi, with similar severity at these time points (Fig 1A). Initially, we used the NORT to test cognition (learning and memory recall). The ability to distinguish new and familiar objects was preserved during the acute infection (5–9 dpi) and after parasite control (82–86 dpi). However, this ability was reduced, revealing impairment in object recognition memory when parasitemia is intermittent or not apparent at 120 and 150 dpi (Fig 1A).

Further, we tested spatial habituation memory by counting the total crossed lines in the two days of exposition to the open field (OFT2). Interestingly, the ability to recognize the known environment was preserved from 5 dpi up to 120 dpi, while this spatial habituation memory was disrupted at 150 dpi in all infected mice (Fig 1A). Therefore, our results demonstrate that *T*. *cruzi* infection sequentially undermines behavioral and cognitive traits: firstly, reduced innate repetitive, compulsive behavior; secondly, anxiety-like behavior and depressive-like behavior; lastly, memory deficit prevailed with novel object recognition impairment preceding spatial habituation memory loss.

GMST was performed in chronically infected mice as some behavioral activities require muscle strength. Compared with NI controls, *T*. *cruzi*-infected mice show preserved muscle strength at 120 dpi (S2B Fig). At this time point, in comparison with age-matched NI controls, *T*. *cruzi*-infected mice showed augmented latency to push sawdust toward a marble (*p* < 0.001) and a reduction in the number of hidden spheres (*p* < 0.001) at the MBT (Fig 1B). Anxiety, shown as a decrease in the number of entries and time expended in the open arms of the maze apparatus at the EPMT (*p* < 0.001), was detected in all analyzed *T*. *cruzi*-infected mice at 120 dpi (Fig 1C). At this time point, *T*. *cruzi*-infected mice also exhibited depressive-like behavior, revealed as an increase in immobility in the TST (*p* < 0.001) and FST (*p* < 0.001) (Fig 1D). At 120 dpi, *T*. *cruzi*-infected mice showed a reduced discrimination index of non-spatial habituation memory assessed by the NORT (*p* < 0.05) (Fig 1E). However, the spatial habituation memory evaluated by the number of crossed lines in the open field in the two-day test (OFT2) was similar in both NI and infected mice groups (Fig 1E). Thus, at 120 dpi, the object recognition memory was impaired, while the spatial habituation memory was preserved.

Tryptophan degradation by IDO has important neuropsychiatric implications because tryptophan catabolites may induce neurodegeneration, and tryptophan is a precursor of serotonin [43]. Thus, given that IDO fluctuation in the CNS may affect serotonin and contribute to depression, we analyzed the expression of IDO. Enhanced expression of IDO was detected in the encephalon of acute (35–40 dpi) and chronically (120 dpi) *T*. *cruzi*-infected mice (*p* < 0.01), compared with brains of age-matched NI controls (Fig 1F). SERT/5-HTT is responsible for Ser/5-HT reuptake by presynaptic neurons and glial cells [44]. Thus, alteration in SERT/5-HTT expression may modify Ser/5-HT availability. In the encephalon of acute and chronically infected mice, SERT/5-HTT expression was consistently reduced by an average of 20–25% (*p* = 0.0592, acute *vs* NI; *p* < 0.05, chronic *vs* NI), in comparison with brains of sex- and age-matched NI controls (Fig 1F). Cytokine-driven upregulation of IDO has been associated with behavioral abnormalities [43]. At 120 dpi, IFNγ and TNF mRNA expression were similar in the encephalon of NI controls and *T*. *cruzi*-infected C57BL/6 mice (Fig 1G). However, at this time point, increased concentrations of IFNγ (*p* < 0.001) and TNF (*p* < 0.01) were detected in the serum of *T*. *cruzi*-infected mice, compared with NI controls (Fig 1H).

### Fluoxetine therapy ameliorates behavioral and cognitive changes in chronically *T*. *cruzi*-infected mice and repositions neurochemical abnormalities

**Fig 2:**
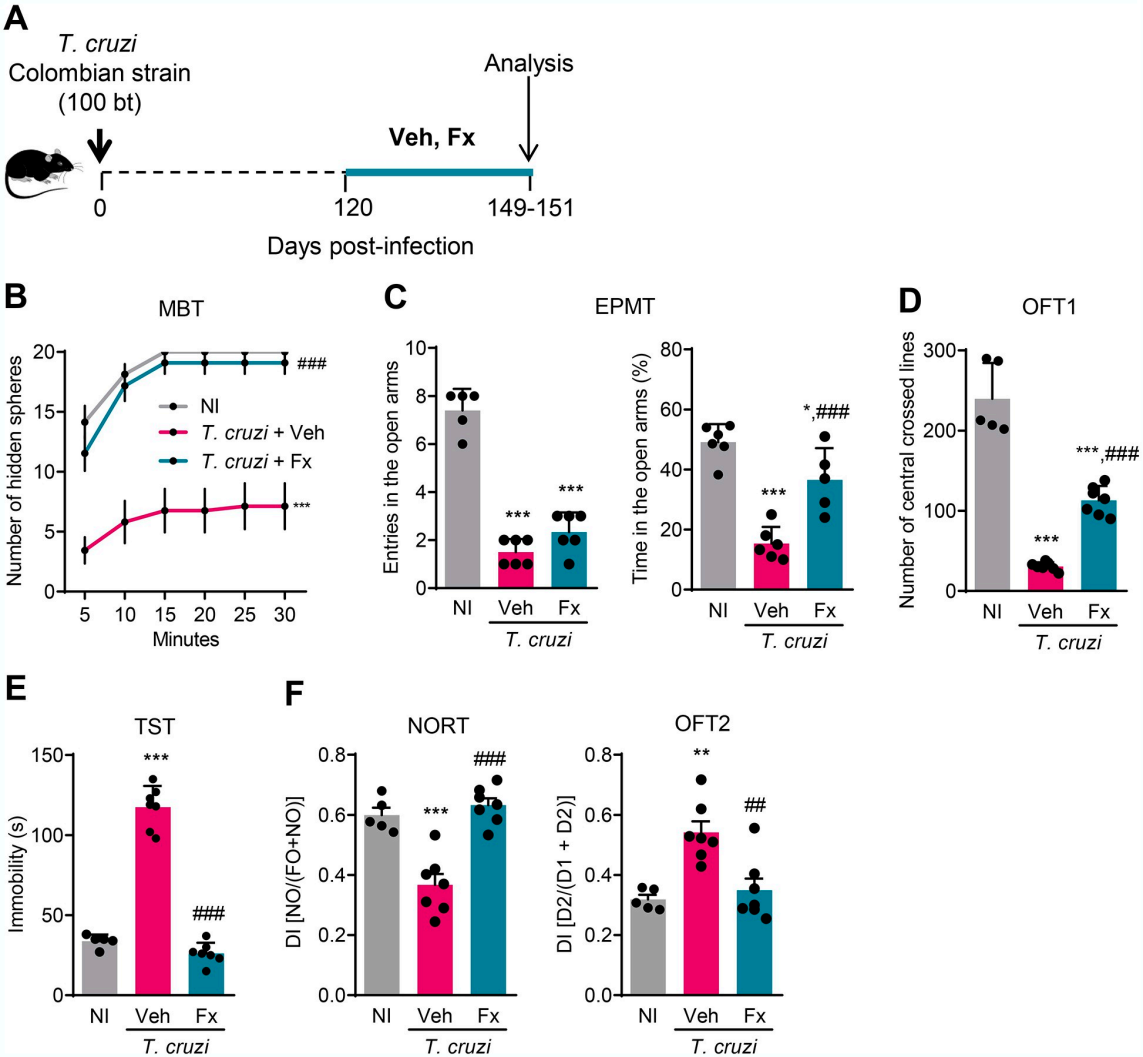
Behavioral alterations in chronically *Trypanosoma cruzi*-infected mice are lessened or reversed by Fx administration. (**A**) Therapeutic scheme: C57BL/6 mice were infected with 100 bt of the Colombian *T*. *cruzi* Type I strain. Initiating at 120 dpi, mice received gavage with Fx therapy (10 mg/Kg/day) or vehicle control (Veh) for 30 consecutive days. At 150 dpi, mice were analyzed using standardized behavioral tests. (**B**) Number of hidden spheres in a 30-minute marble burying test (MBT). (**C**) Number of entries and time expended in the open arms in an elevated plus maze test (EPMT). (**D**) Number of central crossed lines in the open field test (OFT1). (**E**) Time of immobility in the tail-suspension test (TST). (**F**) Discrimination index (DI) in the novel object recognition memory task (NORT) and the two-day exposition to the open field test (OFT2). Results representative of two independent experiments. Color code: Grey bars indicate non-infected controls (NI), pink bars show Veh-treated, and blue bars indicate Fx-treated infected mice. Each dot represents a mouse. The data are shown as the means ± SD. *, *p* < 0.05, **, *p* < 0.01 and ***, *p* < 0.001, *T*. *cruzi*-infected compared with NI controls. ^##^, *p* < 0.01 and ^###^, *p* < 0.001, Fx-treated compared with Veh-treated *T*. *cruzi*-infected mice.

Fx, an SSRI mainly acting on SERT/5-HTT, is used as an antidepressant and may also affect anxiety and cognition [42]. The findings in pre-therapy groups are shown in Fig 1A–1E, supporting that the initiation of treatment at 120 dpi allowed to challenge the putative effects of Fx on preventing the onset or reversing behavioral and cognitive changes in experimental CD. Thus, chronically *T*. *cruzi*-infected C57BL/6 mice were submitted to Fx or Veh for 30 consecutive days from 120 to 150 dpi (Fig 2A). At 150 dpi, all *T*. *cruzi*-infected mice lost the innate compulsive behavior of burying aversive materials (marbles, in the used test) present in their environment (*p* < 0.001), contrasting with the innate behavior detected in NI control mice (Figs 1A and 2B). Compared with Veh administration, Fx therapy recovered the innate compulsive behavior (assessed by MBT) in infected mice (*p* < 0.001; Fig 2B). When submitted to EPMT, Veh-treated infected mice showed a reduced number of entries (*p* <0.001) and time expended (*p* <0.001) in the open arms, indicating anxiety-like behavior. After Fx administration, although the number of entries in the open arms remained reduced (*p* = 0.0646), the time expended in the open arms of the labyrinth was partially restored (*p* < 0.001), in comparison with Veh-treated infected mice (Fig 2C). At 150 dpi, Veh-treated infected mice crossed fewer central lines at the OFT1 than NI controls (*p* < 0.001; Fig 2D), supporting anxiety-like behavior. After Fx therapy, an increment in the number of central crossed lines was observed (*p* < 0.001); however, this was a partially beneficial effect compared with the performance of NI controls (*p* < 0.001). Thus, in chronically *T*. *cruzi*-infected mice, Fx therapy improved but did not resolve anxiety-like behavior. Compared with age- and sex-matched NI controls, Veh-treated infected mice showed increased immobility time in TST (*p* < 0.001) at 150 dpi. As expected, Fx-treated infected mice had a reduced time of immobility (*p* < 0.001), resembling NI control mice (Fig 2E), showing that depressive-like behavior was completely reversed in Fx-treated chronically infected mice.

Controversial effects of Fx on cognition have been described [45]; therefore, we tested the effects of this antidepressant on sequential memory impairments in chronically *T*. *cruzi*-infected mice (Fig 1A). In comparison with age-matched NI controls, infected C57BL/6 mice show loss of object recognition (*p* < 0.001) and spatial habituation memory (*p* < 0.01) at 150 dpi (Fig 2F). Significantly, Fx administration completely reversed the deficit of object recognition memory (*p* < 0.001) and prevented spatial habituation memory impairments (*p* < 0.01; Fig 2F). Therefore, Fx therapy improved mnemonic deficits and hampered progressive memory loss in chronically *T*. *cruzi*-infected mice.

The contribution of heart abnormalities to CNS impairment in CD is a matter of debate [11]. Fx therapy improves heart rate in a non-infectious condition [46]. Thus, we checked whether the amelioration of behavioral and cognitive changes detected after Fx therapy was associated with improved heart abnormalities in chronically infected mice. At 150 dpi, ECG registers showed alterations in infected C57BL/6 mice compared with NI controls (S3A Fig). Indeed, most of the chronically infected mice show arrhythmias (*p* < 0.01) and second-degree atrioventricular blocks (AVB2; *p* < 0.01), as shown in S3B Fig. Further, these mice show bradycardia (*p* < 0.001), prolonged PR interval (*p* < 0.001), and increased dispersion of QTc interval (*p* < 0.01), as shown in S3C Fig. Fx therapy beneficially affected the frequency of mice committed by arrhythmias (*p* < 0.05) but had no effects on the frequency of mice with AVB2 (S3B Fig). Moreover, Fx therapy improved heart rate but did not impact prolonged PR interval and QTc dispersion (S3C Fig). In chronically infected C57BL/6 mice, ECG abnormalities have been associated with increased TNF expression in the heart tissue [47]. Further, TNF modifies Ser/5-HT-SERT biology in non-infectious conditions [45]. At 150 dpi, TNF expression is enhanced in the cardiac tissue and was not impacted by Fx therapy (S3D Fig). Thus, Fx administration to chronically *T*. *cruzi*-infected mice had a discreet effect on ECG abnormalities but no impact on the upregulated TNF expression in the heart tissue. Notably, ECG changes were not aggravated after Fx therapy.

**Fig 3:**
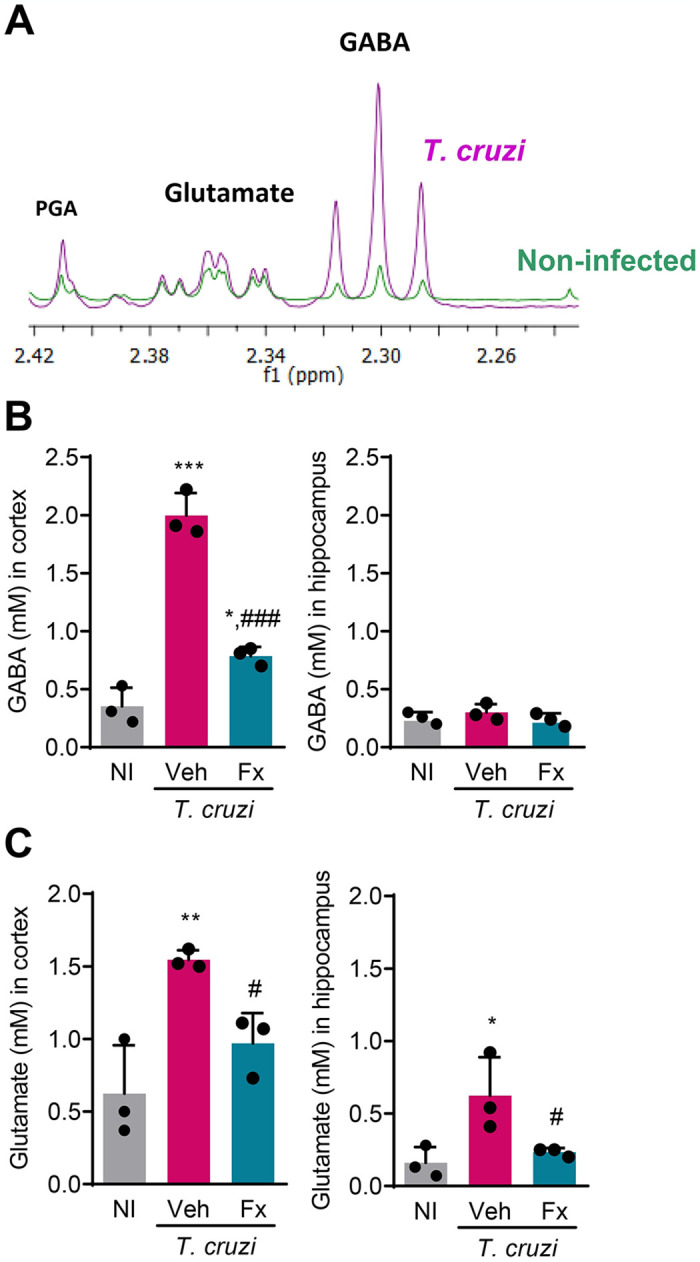
Enhanced levels of neurotransmitters are reduced by Fx administration to chronically *Trypanosoma cruzi*-infected mice. (**A**) Representative profiles of GABA, glutamate, and pyroglutamic acid (PGA) in the brain tissue, analyzed by nuclear magnetic resonance, of non-infected controls (NI) and *T*. *cruzi*-infected mice at 120 dpi. (**B**) GABA and (C) glutamate concentrations in NI controls’ brain cortex and hippocampus areas, and Veh-treated and Fx-treated *T*. *cruzi*-infected mice, at 150 dpi. Representative data of two independent experiments. Color code: Grey bars indicate NI, pink bars show Veh-treated, and blue bars indicate Fx-treated infected mice. Each dot represents a mouse. The data are shown as the means ± SD. *, *p* < 0.05, **, *p* < 0.01 and ***, *p* < 0.001, *T*. *cruzi*-infected compared with NI controls. ^#^, *p* < 0.05 and ^###^, *p* < 0.001, Fx-treated compared with Veh-treated *T*. *cruzi*-infected mice.

Next, we tested whether canonical neurochemical changes are detected in the CNS of chronically *T*. *cruzi*-infected mice and the putative effects of Fx therapy on these abnormalities. At 120 dpi, we detected increased levels of GABA, glutamate, and pyroglutamic acid (PGA) in brain extracts of chronically *T*. *cruzi*-infected C57BL/6 mice, compared with NI controls (Fig 3A). At 150 dpi, increased GABA concentrations were detected in the cortex (*p* < 0.001), but not in the hippocampus, of Veh-injected infected mice, compared with NI controls (Fig 3B). Also, increased concentrations of glutamate were detected in the cerebral cortex (*p* < 0.01) and hippocampus (*p* < 0.05) of Veh-treated infected mice (Fig 3C). Significantly, Fx therapy reduced GABA concentrations (*p* < 0.001) in the cerebral cortex (Fig 3B) and glutamate concentrations in the cortex (*p* < 0.05) and hippocampus (*p* < 0.05) of chronically infected mice (Fig 3C).

**Fig 4:**
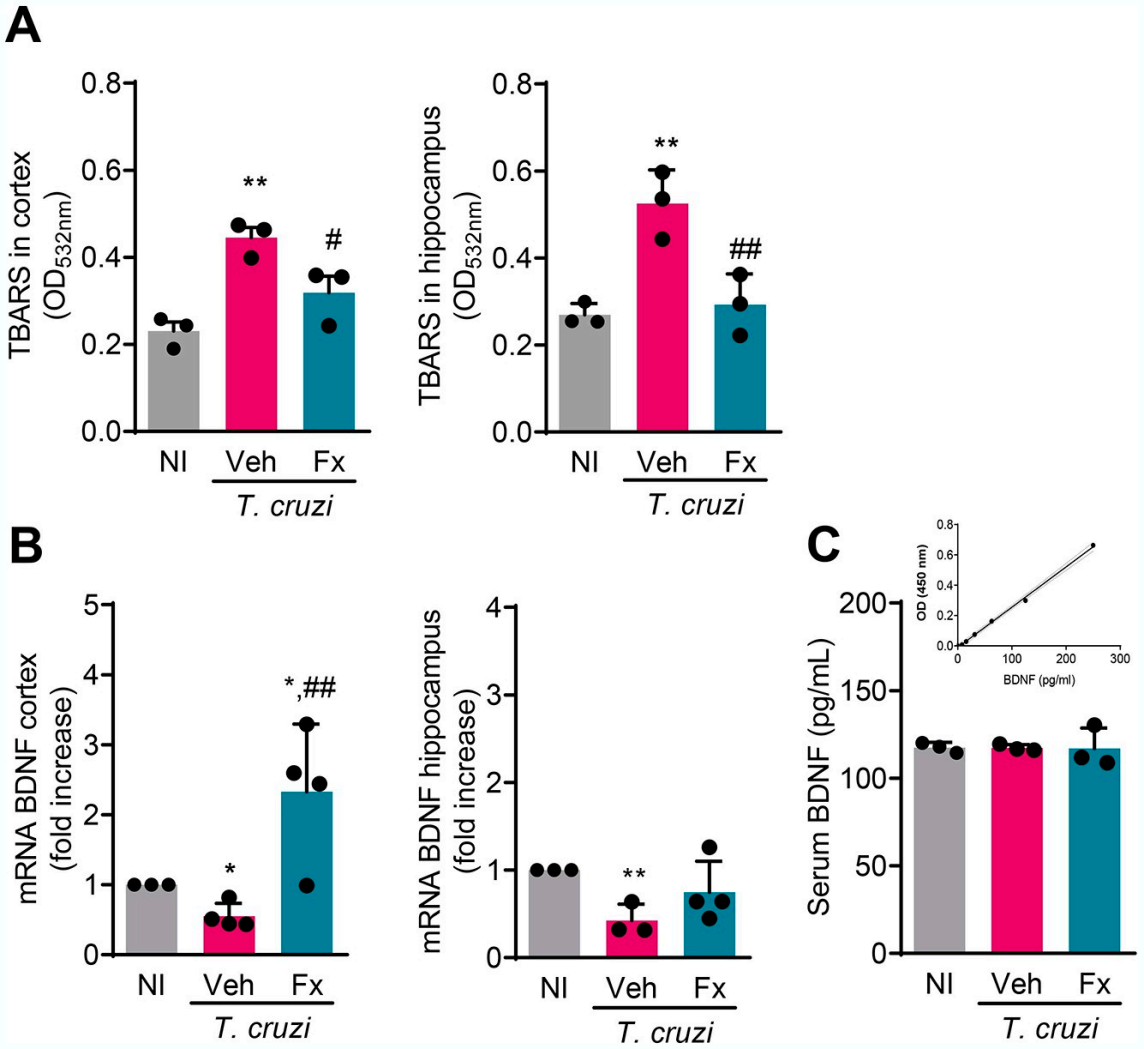
Fx therapy ameliorates oxidative stress and BDNF expression in chronically *Trypanosoma cruzi*-infected mice. (**A**) Oxidative stress, revealed by detection of TBARS, and (**B**) BDNF mRNA levels in the brain cortex and hippocampus areas. (**C**) BDNF concentrations in serum of NI controls and Veh- and FX-treated *T*. *cruzi*-infected mice, at 150 dpi. Representative data of two independent experiments. Color code: Grey bars indicate non-infected controls (NI), pink bars show Veh-treated and blue bars indicate Fx-treated infected mice. Each dot represents a mouse. The data are shown as the means ± SD. *, *p* < 0.05 and **, *p* < 0.01, *T*. *cruzi*-infected compared with NI controls. ^#^, *p* < 0.05 and ^##^, *p* < 0.01, Fx-treated compared with Veh-treated *T*. *cruzi*-infected mice.

At 150 dpi, oxidative stress revealed as increased TBARS levels [22], was detected in the cerebral cortex (*p* < 0.01) and hippocampus (*p* < 0.01) of Veh-injected *T*. *cruzi*-infected mice, compared with NI controls (Fig 4A). Significantly, Fx therapy reduced TBARS levels in the cortex (*p* < 0.05) and hippocampus (*p* < 0.01) of infected mice (Fig 4A). Hence, Fx therapy reduced the levels of the pan-marker of oxidative stress TBARS in the CNS areas of chronically *T*. *cruzi*-infected mice.

The neurotrophin BDNF plays a role in crucial processes of learning and memory formation [48]. At 150 dpi, compared with NI controls, a decrease in BDNF expression was observed in the cerebral cortex (*p* < 0.05) and hippocampus (*p* < 0.01) of Veh-injected *T*. *cruzi*-infected C57BL/6 mice (Fig 4B). Fx therapy increased BDNF expression in the cortex region of infected mice, compared with Veh-treated (*p* < 0.01). Fx therapy increased BDNF expression in the cortex compared with NI controls (*p* < 0.05). Fx therapy tended to normalize BDNF expression in the hippocampus of chronically infected mice (Fig 4B). BDNF serum levels are reduced in depressive disorder [49]. Here, we showed that BDNF is detected in the serum of NI control mice. However, neither chronic *T*. *cruzi* infection nor Fx administration impacted circulating BDNF concentrations (Fig 4C).

**Fig 5:**
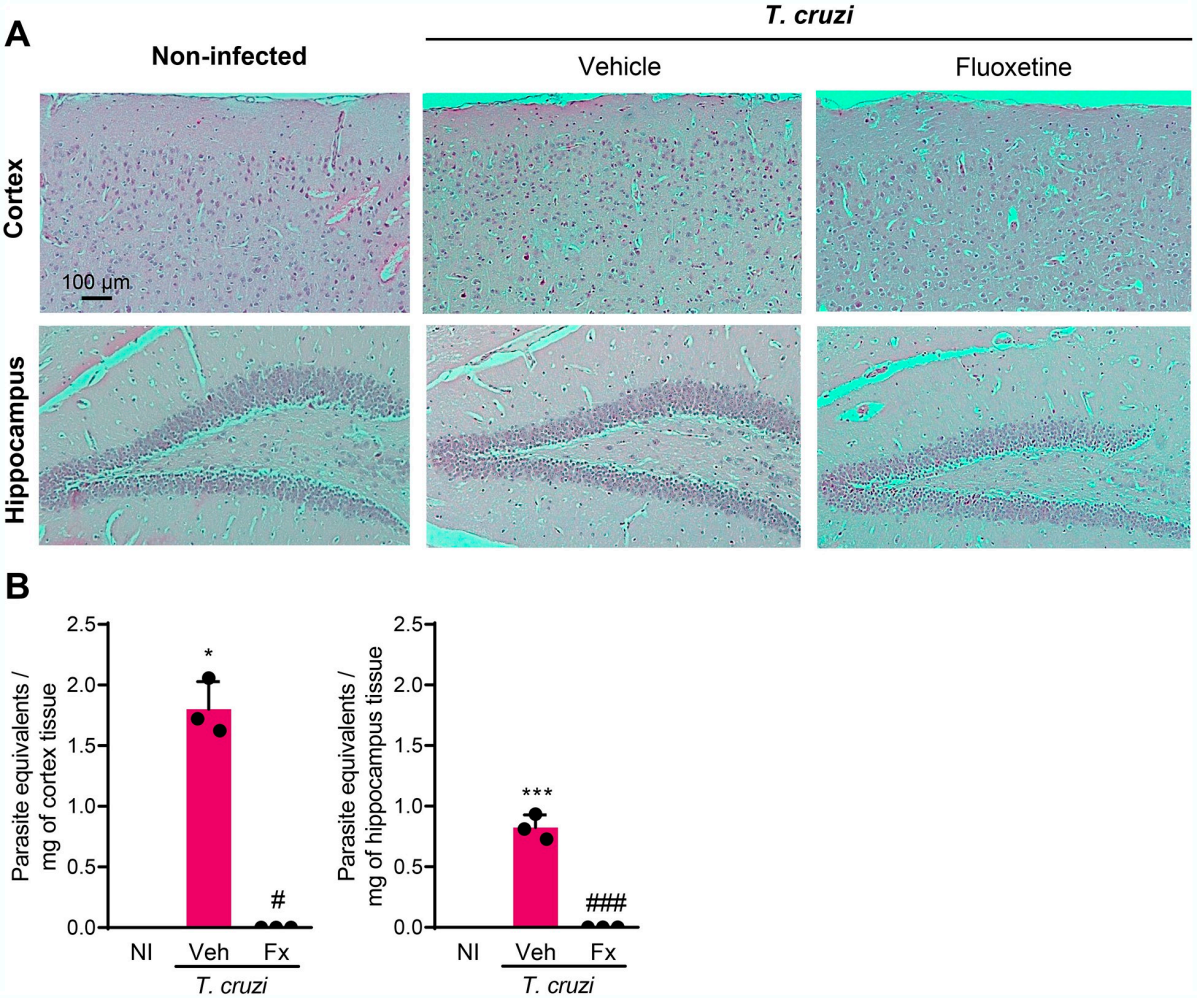
Fx treatment reduces parasite load in brain areas of chronically *Trypanosoma cruzi*-infected mice. (**A**) Histological sections of the cortex and hippocampus of non-infected controls (NI) and chronically *T*. *cruzi*-infected mice at 150 dpi. (**B**) Parasite DNA quantified by qPCR in the cortex and hippocampus of NI and infected mice, at 150 dpi. Representative data of two independent experiments. Color code: grey bars indicate NI, pink bars show Veh-treated and blue bars indicate Fx-treated infected mice. Each dot represents a mouse. The data are shown as the means ± SD. *, *p* < 0.05 and ***, *p* < 0.001, *T*. *cruzi*-infected compared with NI controls. ^#^, *p* < 0.05 and ^###^, *p* < 0.001, comparing Fx-treated with Veh-treated *T*. *cruzi*-infected mice.

Neuroinflammation, manifested by invasion of brain tissue by inflammatory cells [24], is not detected in the CNS of chronically *T*. *cruzi*-infected C57BL/6 mice [22]. Here, we confirmed the absence of neuroinflammation in the cortex and hippocampus of *T*. *cruzi*-infected mice at 150 dpi (Fig 5A). Further, Fx therapy did not induce inflammation or architectural alterations in the CNS (Fig 5A). Lastly, we tested the effects of Fx therapy on parasite load in the CNS. Parasite DNA was detected by qPCR in the cerebral cortex (*p* <0.05) and hippocampus (*p* <0.001) of chronically *T*. *cruzi*-infected C57BL/6 mice (Fig 5B). Surprisingly, Fx therapy reduced parasite load in the cerebral cortex (*p* <0.05) and hippocampus (*p* <0.001) in comparison with Veh administration (Fig 5B). Therefore, these findings suggest that Fx may have a direct action in controlling the parasite in CNS cells, or the Ser/5-HT pathway may play a role in the infection of CNS cells by *T*. *cruzi*, opening new avenues to add mechanistic insights into host/parasite interactions.

### *T*. *cruzi* infection and production of neuromediators are fueled by serotonin and cytokines stimulation but controlled by fluoxetine treatment of the human U-87 MG glial cell lineage

**Fig 6:**
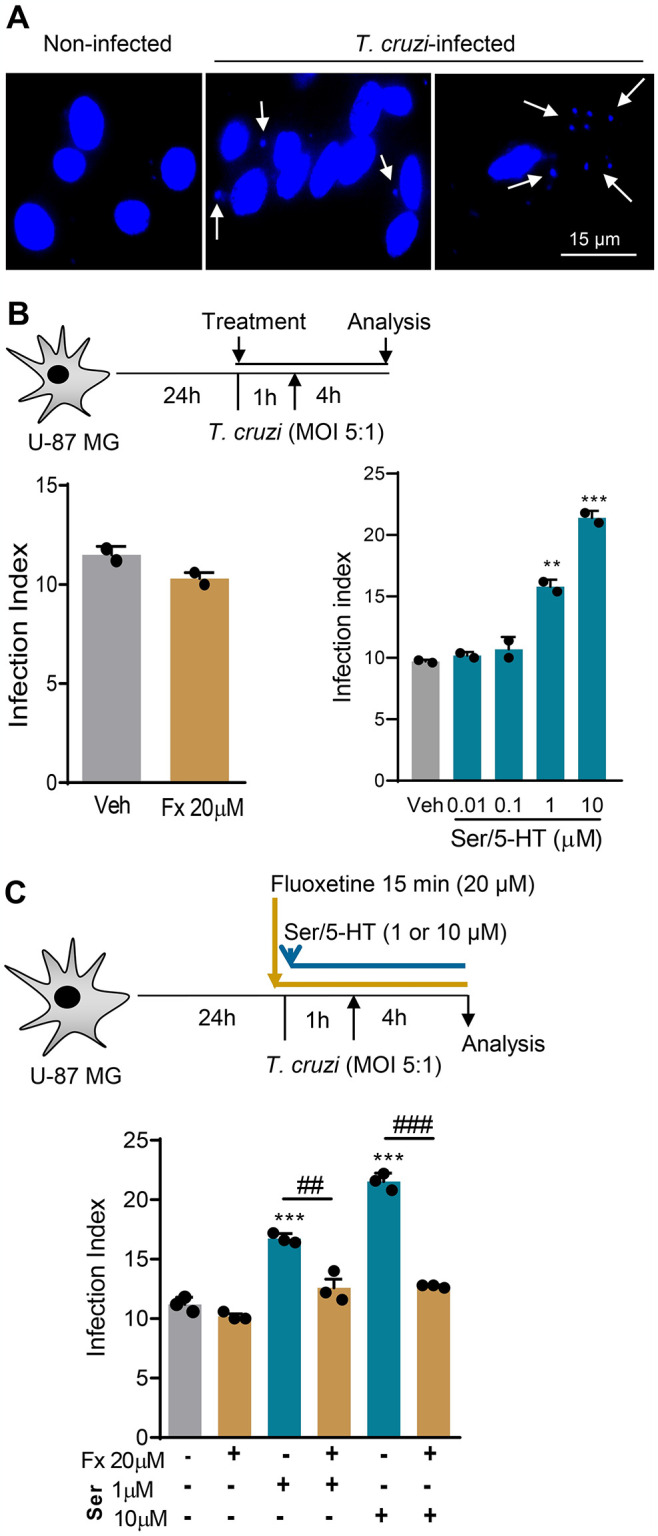
Fx treatment abrogates the Ser/5-HT-promoted *Trypanosoma cruzi* infection of human U-87 MG astroglioma cells. (**A**) Representative pictures of DAPI-labelled DNA of cells U-87 MG (large blue nucleus) and parasites (small blue nucleus/kDNA), showing the presence of intracellular parasites (arrows), 4 hours postinfection. (**B**) The scheme shows that cultures of U-87 MG cells were settled, treated with Fx or Ser/5-HT, infected with cell culture-derived trypomastigote forms of the Colombian *T*. *cruzi* strain using a multiplicity of infection (MOI) of 5:1 (parasite: cell) and interaction periods of 4 hours. The graph shows the infection index induced by Fx (20 μM) or Ser/5-HT (0.01 to 10 μM), compared with vehicle-treated (Veh) cells. (**C**) The scheme shows that cultures of U-87 MG cells were settled, treated with Fx, stimulated with Ser/5-HT, infected with trypomastigote forms of the Colombian *T*. *cruzi* strain (MOI of 5:1), and analyzed 4 hours later. The graph shows the infection index induced by Ser/5-HT (1 and 10 μM) in the absence or presence of Fx (20 μM), at 4 hours postinfection. Two or three independent experiments were performed. Each dot represents the data of an independent experiment performed in triplicate. The data are shown as the means ± SD. **, *p* < 0.01 and ***, *p* < 0.001, Ser/5-HT-stimulated compared with not stimulated infected cells. ^##^, *p* < 0.01 and ^###^, *p* < 0.001, comparing Fx-treated with not treated Ser/5-HT-stimulated *T*. *cruzi*-infected cells.

In mice, astrocytes are the main auberge of *T*. *cruzi* in the CNS in the acute and chronic phases of infection [50]. To investigate the effect of Fx therapy on parasite control in the CNS, we used the human U-87 MG astroglioma cell lineage, a model to study astrocyte-like cell biology [51]. Firstly, we showed that the addition of Fx (1 to 100 μM) or Ser/5-HT (1 to 1000 μM) to U-87 MG cell cultures did not impact cell viability (see S4 Fig). U-87 MG cells are susceptible to infection with cell culture-derived trypomastigote forms of the Colombian strain [41]. U-87 MG cell cultures were settled, and 24 hours later, Fx was added 1 hour before infection (Fig 6A). Fx had no direct effect on infection control, compared with the addition of Veh (Fig 6B). Then, considering that U-87 MG is responsive to Ser/5H-T [40], we tested whether this neurotransmitter may favor infection. Cell cultures were stimulated with Ser/5-HT (0.01 to 10 μM) 1 hour before infection (Fig 6B). Notably, the stimulus of U-87 MG cells with 1 μM (*p* <0.01) and 10 μM (*p* <0.001) of Ser/5-HT enhanced the infection index in comparison with the addition of Veh (Fig 6B). Fx binds to SERT/5-HTT, blocking Ser/5-HT reuptake by presynaptic neurons and glial cells [44]. Therefore, we tested the effect of Fx on infection and Ser/5-HT-promoted infection of U-87 MG cells by *T*. *cruzi* parasites (Fig 6C). Fx (20 μM) did not affect the basal condition of U-87 MG cells by cell culture-derived trypomastigote forms of the Colombian strain (Fig 6B and 6C). However, the addition of Fx before Ser/H-5T stimulus abrogated the effect of 1 μM (*p* <0.01) and 10 μM (*p* <0.001) of Ser/5-HT fueling infection of U-87 MG cell by *T*. *cruzi* (Fig 6C).

**Fig 7:**
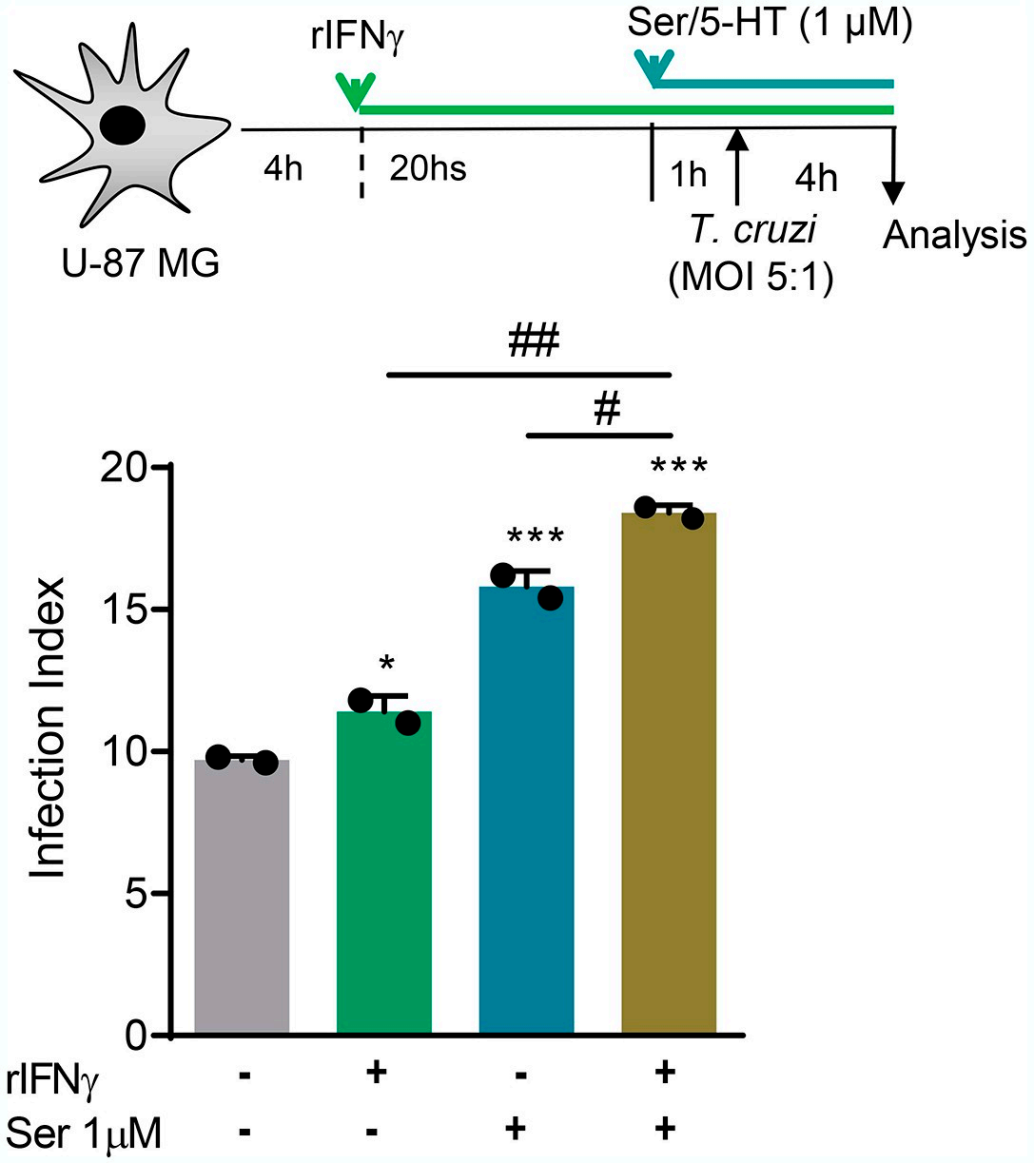
IFNγ enhances the Ser/5-HT-promoted *Trypanosoma cruzi* uptake by the human U-87 MG astroglioma cells. The scheme shows that cultures of U-87 MG cells were settled and treated with recombinant human IFNγ (rIFNγ, 10 ng/mL) for 20 hours; Ser/5-HT was added (1 μM), 1 hour later, cultures were infected with cell culture-derived trypomastigote forms of the Colombian *T*. *cruzi* strain (MOI of 5:1) and analyzed 4 hours later. The percentage of infected cells and the number of intracellular forms were counted to determine the infection index (Infection Index = percentage of infected cells x number of parasites per infected cell). The graph shows the infection index of two independent experiments. Each dot represents the data of a separate experiment performed in triplicate. The bars show the means ± SD. *, *p* < 0.05 and ***, *p* < 0.001, experimental conditions compared with vehicle-treated infected cells. ^#^, *p* < 0.05 and ^##^, *p* < 0.01, comparing the double-treated (rIFNγ and Ser/5-HT) cells with cells subjected to a single treatment (rIFNγ or Ser/5-HT).

IFNγ increases *in vitro* infection of murine astrocytes by trypomastigote forms of *T*. *cruzi* [50]. Thus, we tested whether recombinant human IFNγ (rIFNγ) influences Ser/5-HT effects on infection of U-87 MG cells by *T*. *cruzi*. U-87 MG cultures were treated with rIFNγ (10 ng/mL) before adding Ser/5-HT (1 μM), followed by *T*. *cruzi* infection. The rIFNγ treatment of U-87 MG cells augmented the infection of these cells by trypomastigote forms (*p* < 0.05). Moreover, pretreatment with rIFNγ increased (*p* < 0.05) the effects of Ser/5-HT promoting the infection of U-87 MG cells by *T*. *cruzi* (Fig 7).

**Fig 8:**
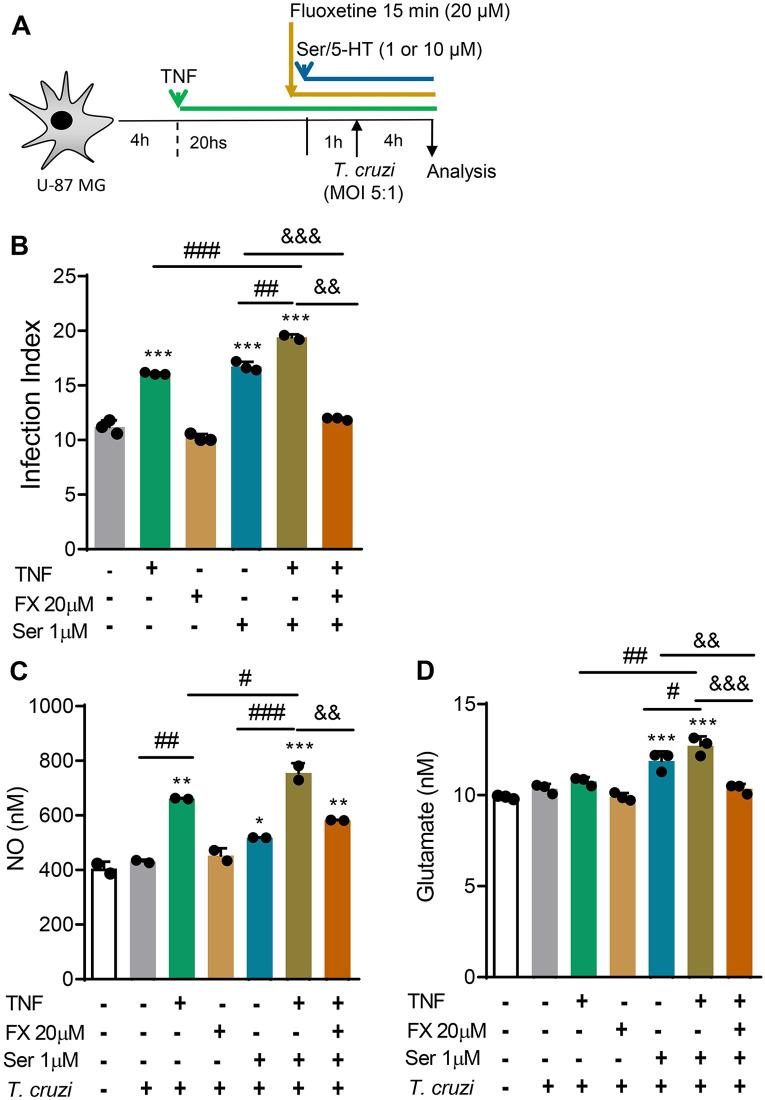
Fx controls the TNF-enhanced Ser/5-HT-promoted *Trypanosoma cruzi* uptake by U-87 MG cells and neuromediators availability. (**A**) The scheme shows that cultures of U-87 MG cells were settled, treated with recombinant human TNF (rTNF, 10 ng/mL) for 20 hours, Ser/5-HT was added (1 μM), 1 hour later infected with cell culture-derived trypomastigote forms of the Colombian *T*. *cruzi* strain (MOI of 5:1), and analyzed 4 hours later. The percentage of infected cells and the number of intracellular forms were counted to determine the infection index (Infection Index = percentage of infected cells x number of parasites per infected cell). (**B**) The graph shows the infection index in cell cultures submitted to the indicated conditions. (**C**) The graph shows NO concentrations in the supernatant of cultures presented to the indicated conditions. (**D**) The graph shows glutamate concentrations in the supernatant of cultures submitted to the indicated conditions. Each dot represents the data of an independent experiment performed in triplicate. The bars show the means ± SD. *, *p* < 0.05; **, *p* < 0.01 and ***, *p* < 0.001, experimental conditions compared with vehicle-treated infected cells (**A**) or non-infected cells (**C** and **D**). ^#^, *p* < 0.05; ^##^, *p* < 0.01and ^###^, *p* < 0.001, comparing among experimental conditions, as indicated by horizontal lines. ^&&^, *p* < 0.01and ^&&&^, *p* < 0.001, comparing experimental conditions with pre-exposition to Fx, as indicated by horizontal lines.

Our previous data show that IFNγ fuels the infection of mouse and human astrocytes by *T*. *cruzi* via autocrine TNF production [50]. Here, we corroborated this finding, showing that the anti-TNF neutralizing antibody Infliximab (Remicade) completely abrogated the rIFNγ-promoted infection of the human astrocyte U-87 MG cells by *T*. *cruzi* (S5 Fig). TNF increases SERT/5-HTT expression in astrocytes and SERT-mediated Ser/5-HT uptake [52]. Thus, we tested the effect of recombinant human TNF (rTNF) on Ser/5-HT-promoted U-87 MG infection by *T*. *cruzi* and the impact of Fx on these conditions, as shown in the experimental scheme (Fig 8A). Importantly, rTNF (10 ng/mL) nourished the infection of U-87 MG human astrocytes by *T*. *cruzi* (*p* <0.001) and increased the Ser/5-HT-promoted infection (*p* <0.001). Moreover, treatment with Fx completely abrogated (*p* <0.01) the rTNF-increased Ser/5-HT-promoted infection of astrocytes (Fig 8B).

The neuromediators nitric oxide (NO) and glutamate play a role in behavioral and cognitive control [53]. Compared with non-infected cells, the infection of U-87 MG cells by *T*. *cruzi* was insufficient to modify the NO concentrations in supernatants (Fig 8C). However, NO concentrations were increased in the presence of two stimuli: TNF and *T*. *cruzi* (*p* <0.01) or Ser/5H-T and *T*. *cruzi* (*p* <0.05). This effect was even enhanced when the three stimuli were present (*p* <0.05, TNF, Ser/5H-T and *T*. *cruzi vs* TNF and *T*. *cruzi; p* <0.001, TNF, Ser/5H-T, and *T*. *cruzi vs* Ser/5H-T and *T*. *cruzi*) (Fig 8C). The addition of Fx only partially reduced NO concentrations (*p* <0.01, TNF, Fx, Ser/5H-T and *T*. *cruzi vs* TNF, Ser/5H-T, and *T*. *cruzi*), and NO levels remained increased in comparison with non-infected cells (Fig 8C). Glutamate concentrations in the supernatants of U-87 MG human astrocyte cultures were not altered by infection or treatment with TNF before infection, compared with non-infected cells (Fig 8D). However, glutamate levels were enhanced in the presence of Ser/5-HT: Ser/5H-T and *T*. *cruzi* (*p* <0.001) or TNF, Ser/5H-T and *T*. *cruzi* (*p* <0.001). The addition of Fx normalized the concentrations of glutamate to levels detected in non-infected cells (*p* <0.01, TNF, Fx, Ser/5H-T and *T*. *cruzi vs* Ser/5H-T, and *T*. *cruzi*; *p* <0.001, TNF, Fx, Ser/5H-T and *T*. *cruzi vs* TNF, Ser/5H-T and *T*. *cruzi*), as shown in Fig 8D.

## Discussion

Unprecedentedly, we showed the sequential appearance of behavioral and cognitive disorders in an experimental model of acute and chronic CD, linking these changes to key canonical biomarkers and offering therapeutic insights. Our data support that a complex interplay between neurotransmitters and cytokines favors the persistence of *T*. *cruzi* within the CNS in the chronic phase of infection. Lastly, we outlined a schematic proposal for the CNS involvement in *T*. *cruzi* infection.

Patients with CD may experience poor quality of life, headache, confusion, speech disorders [13,16], sleep dysfunction [17], anxiety, depression [14,15,19], attention deficits, cognitive and mnemonic deficits [12,13,17], traits that may co-exist. Low socio-economic status and psychological stressors may contribute to these mental disorders in CD patients [8,54]. However, the presence of behavioral changes such as sleep disturbance [20], anxiety, depressive-like disorders [21, 23], and memory loss [22] in experimental models of CD support a role for biological stressors in these processes. Here, we present the first kinetic study that supports the sequential onset of behavioral and cognitive changes in Colombian-infected C57BL/6 mice. Trying to establish a relationship between these behavioral changes and biomarkers of *T*. *cruzi* infection, we picked up time points before and after the peak of parasitemia, when parasitemia is controlled and in the chronic phase, as well as time points related to the onset of heart disease and critical immune response traits in this CD model [30,55]. Here, the initial loss of an innate behavior was followed by anxiety and depressive-like behavior, ending in progressive memory loss, corroborating the sequential onset of object recognition, spatial habituation, and aversive memory deficits described in chronically infected mice [22]. Thus, our study in infectious disease may support the network theory, which proposes that an initial mental disorder may trigger other processes and act as a comorbid factor, opening a path of progressive changes, as the sequential sleep disorders, anxiety, and depression described in adolescents [56]. In *T*. *cruzi*-infected mice, the sequential onset and simultaneous presence of behavioral and cognitive changes may reveal sequential, parallel, and/or interactive mechanisms underlying these disorders. Although our initial surveys addressed the acute and chronic phases of infection, comprehensive analyses have focused on the chronic phase due to economic limitations and the fact that most CD patients are diagnosed at this phase [10]. At 120 dpi, all behavioral and cognitive alterations assessed are already established, except the spatial habituation memory deficit. Initially, we addressed canonical pathways that may support depressive-like behavior and memory loss [45,53]. Neuroinflammation, defined as the influx of blood-borne inflammatory cells into perivascular cuffs and brain parenchyma [24], and cytokines produced in the CNS are recognized for their roles in behavioral changes in infectious [11,36] and neurodegenerative diseases [24]. In our model of chronic CD, inflammation is absent in the cortex and hippocampus, corroborating previous studies in Colombian-infected C57BL/6 mice [22]. Although we did not detect IFNγ and TNF expression in the CNS of chronically infected mice, their elevated serum levels suggest systemic inflammatory processes. Indeed, systemically elevated levels of IFNγ and TNF have been linked to depressive behavior and memory deficits in infectious [11] and neurodegenerative [57] diseases. Interestingly, anti-TNF monoclonal antibody therapy inhibited depressive-like behavior in experimental chronic CD [21]. Cytokine-driven processes increase IDO expression, shifting the tryptophan pathway towards kynurenine and quinolinic acid, contributing to neuronal cell death and brain atrophy [53]. Fatefully, atrophy of the cerebral cortex, independent of neuroinflammation and cardiac disease, has been detected in patients with CD [58,59] and chronically infected mice [22]. Thus, brain atrophy may reveal the loss of neurons and glial cells, underlying progressive CNS degeneration, and the sequential behavioral and cognitive deficits seen in *T*. *cruzi* infection, an issue to be further explored.

The complexity of depression extends beyond Ser/5-HT imbalance [60], implicating factors such as diet and gut microbiota [61]. However, IDO is crucial for depression by modifying the tryptophan pathway and decreasing the availability of Ser/H-5T, a pleiotropic neurotransmitter that controls physiological, behavioral, and cognitive processes [53,62]. In *T*. *cruzi*-infected mice, an increase in IDO expression in the CNS was detected in the acute phase (35–40 dpi), preceding the initial detection of anxiety and depression (at 82–96 dpi), and persisted in the chronic phase (120 dpi). Elevated IDO expression in the spleen and muscle of acutely *T*. *cruzi*-infected mice has been related to resistance to infection [63]. Here, a consistent decrease of 20–25% of SERT/5-HTT expression was detected in the CNS of acute and chronically *T*. *cruzi*-infected mice, suggesting an imbalance of Ser/5-HT availability. Fx acts primarily on SERT/5-HTT, blocking Ser/5-HT reuptake by presynaptic neurons and glial cells, increasing Ser/5-HT availability in the synaptic cleft [44]. Besides the antidepressant action, Fx may also have effects on anxiety and cognition [28]. Here, we bring the first evidence that the daily four-week Fx regimen effectively reversed depression, as expected, partially ameliorated anxiety while restoring the loss of innate compulsive behavior and deficit of object recognition memory. Moreover, Fx therapy prevented the onset of spatial habituation memory impairment. In an Aβ oligomer-induced model of Alzheimer disease, Fx therapy decreased depression and memory deficit [64]. Fx promotes neuroplasticity by increasing hippocampal synapses and neurogenesis [29], which could underlie its beneficial effects in *T*. *cruzi*-infected mice, an issue to be addressed.

Previous data show that Fx therapy improves heart rate changes in a non-infectious model of chronic mild stress associated with cardiovascular abnormalities [46]. Chronically *T*. *cruzi*-infected mice show bradycardia [30 37], which was improved by Fx therapy; however, relevant aspects of ECG alterations in CD were not modified by Fx treatment, persisting the high frequencies of arrhythmias and AVB2, and the prolonged PR and QTc intervals. In Colombian-infected C57BL/6 mice, elevated expression of TNF in the heart tissue is critically related to chronic heart disease [47]. Here, we used TNF expression in cardiac tissue as a fingerprint of the systemic inflammatory profile, showing the lack of impact of Fx therapy on TNF expression. Therefore, our data support the absence of a correlation between depression and other behavioral changes with heart disease in chronic *T*. *cruzi* infection. Indeed, depression is also detected in CD patients with preserved cardiac function, and positive serology for *T*. *cruzi* emerges as a crucial determinant of this behavioral change [15].

Fx therapy effectively reduced the elevated concentrations of GABA and glutamate in the cortex and hippocampus in chronically infected mice. In a model of chronic restriction, Fx therapy reduced glutamate levels in the CNS and depression [65]. Here, the beneficial effects of Fx reducing GABA and glutamate levels paralleled the resolution of innate compulsive behavior, depression, and memory deficits while improving anxiety, thus suggesting that common biological stressors may contribute to these processes in *T*. *cruzi* infection. Oxidative stress has been associated with memory dysfunction and neurodegenerative disorders [25]. In chronically *T*. *cruzi*-infected mice elevated oxidative stress is detected in the brain tissue [22] and systemically [66]. In this context, the effect of Fx therapy reducing the levels of TBARS in the CNS of chronically *T*. *cruzi*-infected mice, reinforces the contribution of oxidative stress to behavioral and cognitive changes. Oxidative stress and lipid peroxidation can down-regulate neurotrophins such as BDNF [67]. In chronically *T*. *cruzi*-infected mice, the reduced *bdnf* mRNA expression in the CNS was significantly increased in the cortex and normalized in the hippocampus, after Fx therapy. These data support that the idea that the deficit in BDNF is a critical stressor in behavioral changes in *T*. *cruzi* infection. In this condition, Fx may have a broader beneficial effect since recovering BDNF expression is pivotal in managing depression [26] and memory formation [48]. High baseline serum BDNF levels may predict response to antidepressant therapies [49]. In chronically *T*. *cruzi*-infected mice, serum BDNF levels remained unaltered, which may explain the effects of a 4-week Fx therapy, improving depression, anxiety, and memory deficits. In a SERT/5-HTT-independent way, Fx upregulates BDNF expression in mice subjected to chronic stress [68], enhances the phosphorylation of the BDNF tropomyosin receptor kinase B (TrkB), increasing BDNF release, and activates pathways that promote neuronal plasticity and resilience [28, 69]. Thus, these multifaceted actions and other undisclosed pleiotropic roles of Fx may explain the beneficial effects of this antidepressant on behavioral and cognitive changes in chronic *T*. *cruzi* infection.

Parasite forms of *T*. *cruzi* are detected in the CNS of patients, and parasite DNA in tissues revealed the persistence of *T*. *cruzi* in the chronic phase [11]. The antiprotozoal drug benznidazole reduces parasite burden in the CNS, in parallel with reducing oxidative stress and improving mnemonic changes [22]. Surprisingly, the *T*. *cruzi* DNA load in the cerebral cortex and hippocampus of chronically infected C57BL/6 mice was reduced by Fx therapy, reinforcing that parasite persistence in the brain tissue plays a role in behavioral changes. In a model of tumor growth under chronic stress, Fx therapy suppressed the kynurenine pathway, enhanced Ser/5-HT levels and cellular immunity involving CD8^+^ T-cells and IFNγ, and improved tumor control [70]. IFNγ^+^CD8^+^ T-cells are crucial to control *T*. *cruzi* infection [30]. Thus, in the absence of lymphocytic infiltration into the CNS, Fx effects may depend on improving the action of CD8^+^ T-cell in peripheral tissues, reducing parasite burden and access to the CNS. This remains a question to be addressed. Altogether, these data led us to hypothesize that parasite persistence in the CNS is a biological hub triggering neurochemical changes linked to behavioral and cognitive changes. Astrocytes are the main auberge of *T*. *cruzi* in the CNS of mice [50]. Thus, to investigate the effect of Fx therapy on parasite control in the CNS, we used the human astroglioma cell line U-87 MG, a model for studying astrocyte biology [51] and *T*. *cruzi*/glial cell interactions [41]. The infection of U-87 MG is not directly affected by Fx. Although Fx and other SSRIs, such as sertraline, can inhibit *T*. *cruzi* growth in the 3T3 fibroblast cell line, a direct effect of Fx *in vivo* is not likely due to the low concentrations of Fx achieved [71]. Fx also acts on the BDNF receptor TrkB [28]. *T*. *cruzi* uses a GPI-anchored protein, which acts as a parasite-derived neurotrophic factor (PDNF) mimicking the natural ligands and using the TrkA and TrkC receptors, but not TrkB, to infect neuronal and glial CNS cells such as astrocytes [72]. Thus, the effects of Fx on TrkB do not explain the process of parasite control we are currently describing. Fx binds to SERT/5-HTT, blocking Ser/5-HT reuptake by presynaptic neurons and glial cells and abrogates activation of some Ser/H-5T receptors [44]. Also, astrocytes express the serotonin transporter SERT/5-HTT [40, 73]. Considering that U-87 MG responds to Ser/5-HT [40], we tested whether this neurotransmitter could favor infection. Indeed, Ser/5-HT increased the infection of U-87 MG cells by *T*. *cruzi*, an effect abrogated by Fx. Next, we explored the effects of cytokines on the Ser/5-HT-promoted infection. IFNγ fuels *T*. *cruzi* infection of mouse and human astrocytes through autocrine TNF production and involving TNF/TNFR1 signaling [41]. IFNγ and TNF enhanced the Ser/5-HT-promoted *T*. *cruzi* infection of U-87 MG cells. TNF increases SERT/5-HTT expression in astrocytes and SERT-mediated Ser/5-HT uptake [52]. Crucially, Fx completely abrogated the TNF-increased Ser/5-HT-promoted infection of U-87 MG cells by *T*. *cruzi*. *In vivo*, TNF alters the Ser/5-HT pathway, upregulating IDO expression and favoring the production of kynurenine and quinolinic acid [45]. Although we did not detect upregulation of TNF and IFNγ in the CNS of *T*. *cruzi*-infected mice, blood-borne cytokines and other inflammatory mediators may leak into the CNS through areas of disrupted or incomplete blood-brain barrier, such as the choroid plexus, and vagus nerve [74]. These cytokines may activate astrocytes, microglial cells, and neurons, creating a locally constrained, self-sustained inflammatory milieu. In infected mice, IFNγ-producing IBA1^+^microglial cells surround astrocytes harboring *T*. *cruzi* [50]. Therefore, this restricted milieu may amplify Ser/5-HT-promoted astrocyte infection by *T*. *cruzi*, which might be favored by the inability of mouse and human cortical astrocytes (GFAP^+^ cells) to express the inflammasome NPRL3 [75,76]. In contrast, IBA1^+^NPRL3^+^ microglial cells effectively control *T*. *cruzi* infection [75]. Thus, IFNγ may trigger TNF production by astrocytes, which may self-sustain the inflammatory milieu, amplifying the Ser/5-HT-promoted infection of astrocytes by *T*. *cruzi*.

**Fig 9:**
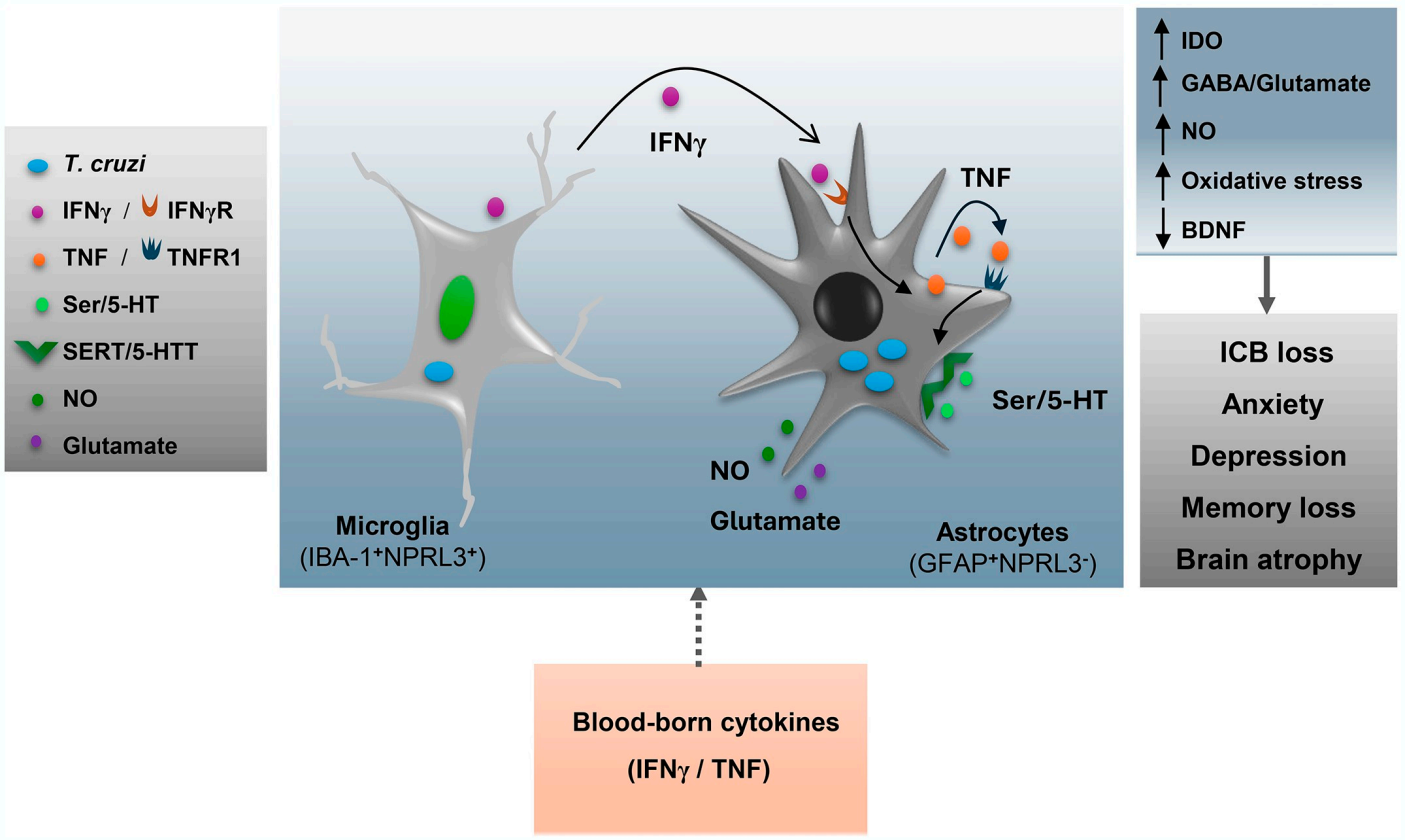
Schematic proposal for mechanisms that induce behavioral and cognitive changes in *Trypanosoma cruzi* infection. In chronic *T*. *cruzi* infection, the input of the blood-borne cytokines IFNγ and TNF into the CNS (dotted lines) may activate glial cells (microglia, IBA-1^+^NPRL3^+^; and astrocytes, GFAP^+^NPRL3^-^). Activated microglia may produce IFNγ. FNγ, acting via IFNγR, may trigger a self-sustaining production of TNF. TNF, acting via TNFR1, may upregulate SERT/5-HTT expression and increase the consumption of Ser/5-HT, fueling astrocyte infection by *T*. *cruzi*. Also, the cytokine-enriched milieu may upregulate the expression of the tryptophan-catabolizing enzyme indoleamine 2,3-dioxygenase (IDO), reducing Ser/5-HT availability. In this scenario, other neurochemical changes may occur, such as (i) augmented levels of the neuromediators nitric oxide (NO), glutamate and gamma-aminobutyric acid (GABA); (ii) increased oxidative stress; and (iii) reduced expression of the neurotrophin brain-derived neurotrophic factor (BDNF). Together, parasite persistence and neurochemical changes may trigger behavioral and cognitive abnormalities, such as innate compulsive behavior (ICB) loss, anxiety, depression, and memory loss, processes occurring in the presence of brain atrophy.

Lastly, we assessed NO and glutamate levels by evaluating the effect of TNF amplification of the Ser/5-HT-promoted increase in *T*. *cruzi* infection of U-87 MG astrocyte cells. NO and glutamate play a role in the control of behavior and cognition [53]. While *T*. *cruzi* infection of U-87 MG cells did not affect NO levels, the combination of TNF plus *T*. *cruzi* or Ser/5-HT plus *T*. *cruzi* increased NO levels, which were even higher in the presence of the three stimuli (TNF, Ser/5-HT plus *T*. *cruzi*), effects only partially inhibited by Fx. Thus, these data place TNF as a pivotal trigger for increasing NO release by *T*. *cruzi*-infected astrocytes. Interestingly, infection alone or TNF stimulation before the infection did not alter the baseline glutamate levels in U-87 MG cell supernatants. Moreover, Ser/5-HT stimulation increased glutamate levels in the presence of *T*. *cruzi* infection or TNF stimulation. Crucially, in this astrocyte/parasite interaction model, Ser/5-HT stimulation was required to increase glutamate levels, and Fx efficiently prevented it. One cannot rule out that, in this case, Fx may act as an anti-inflammatory mediator inhibiting NF-κB activation [77]. Thus, these *in vitro* data may explain our *in vivo* findings. In summary, Fx therapy showed to improve long-lasting behavioral changes and progressive memory loss in an infectious disease for the first time. Further, these beneficial effects of Fx allowed the identification of biological stressors potentially underlying these changes in a model of chronic CD. Indeed, Ser/5-HT signaling fuels the infection of astrocytes by *T*. *cruzi*, a process potentiated by IFNγ and TNF and linked to a neurotoxic milieu enriched in NO and glutamate. Together, cytokine-driven Ser/5-HT consumption may reduce Ser/5-HT availability and favor parasite persistence, creating a neurotoxic milieu and dysregulating neurotransmitter and neurotrophin networks in the CNS, thus contributing to behavioral and cognitive disorders, as schematically proposed in Fig 9.

Age-related physiological deterioration may exacerbate multiple pathogenic processes, priming the brain for neurodegeneration [57]. CD is primarily a stable infectious disease, and clinical signs are detected 10–30 years after infection as people age [10]. Thus, we propose that as *T*. *cruzi* infection progresses, chronic systemic inflammation, oxidative stress, and neurotransmitter alterations may trigger or accelerate aging-related processes in the CNS, opening a sequential onset of behavioral and neurocognitive disorders. In addition to favoring canonical pathways via Ser/5-HT reuptake, increase in BDNF, and antioxidant defenses, Fx could control parasite growth in the CNS, improving behavioral and cognitive changes. More broadly, our findings may pave the way for explaining the late-onset behavioral and cognitive changes in other infectious diseases. We hope that continued progress in reducing the burden of infectious diseases that disproportionately affect low-income people will provide a reasonable countervailing force to improve mental well-being among neglected people [54] while reducing overall healthcare costs related to behavioral disorders [78].

## Supporting information

S1 Fig Workflow of the experimental protocol. The number of C57BL/6 mice non-infected (NI) and infected with 100 bt forms of the Colombian Type I strain (*T*. *cruzi*) used to assess the behavioral and cognitive profiles as a kinetic study (5 to 150 days postinfection, dpi) and to study the effects of vehicle (Veh) and fluoxetine (Fx) administration on the analyzed parameters, including behavioral, and cognitive tests, and electrocardiographic (ECG) registers. Three independent experiments were performed.
(TIF)

S2 Fig Assessment of innate compulsive behavior and muscle strength in *T*. *cruzi*-infected C57BL/6 mice. Non-infected controls (NI) and mice infected with 100 bt forms of the Colombian Type I strain (*T*. *cruzi*) were submitted to (**A**) marble burying test (MBT) from 5–9 up to 82–89 days postinfection (dpi), and (**B**) a non-invasive method to assess the strength of the muscle of mice limbs using grip strength meter test (GMST), at 120 dpi. Color code: (**A**) Grey lines indicate NI controls, and blue lines *T*. *cruzi*-infected mice. (**B**) Grey bars indicate normal values compared with NI controls. (**A**) Each dot represents the means of buried marble at the analyzed moment. (**B**) Each dot represents a mouse. The data are shown as the means ± SD. **, *p* < 0.01 and ***, *p* < 0.001, *T*. *cruzi*-infected compared with NI controls.
(TIF)

S3 Fig Electrocardiographic study and TNF expression in the heart tissue of Fx-treated *T*. *cruzi*-infected C57BL/6 mice. Non-infected controls (NI) and mice infected with 100 bt forms of the Colombian Type I strain (*T*. *cruzi*) were submitted to an electrocardiographic (ECG) study and TNF expression in the heart tissue at 150 days postinfection (dpi). (**A**) Representative ECG register segments of NI and *T*. *cruzi*-infected mice treated with vehicle (Veh) or fluoxetine (Fx) from 120–150 dpi. (**B**) Frequency of mice afflicted by arrhythmias and atrioventricular blocks (AVB2). Each dot represents the percentage of afflicted mice in one independent experiment. (**C**). Average heart rate (HR; beats per minute, bpm), Group data showing PR interval (ms) and dispersion of the QTc interval (ms). (**D**) TNF mRNA expression in the heart tissue at 150 dpi. (**C-D**) Each dot represents a mouse. Results representative of two independent experiments. Color code: Grey bars indicate non-infected controls (NI), pink bars show Veh-treated and blue bars indicate Fx-treated infected mice. The data are shown as the means ± SD. *, *p* < 0.05, **, *p* < 0.01 and ***, *p* < 0.001, *T*. *cruzi*-infected compared with NI controls. ^#^, *p* < 0.05, Fx-treated compared with Veh-treated *T*. *cruzi*-infected mice.
(TIF)

S4 Fig Cell viability of U-87 MG cell cultures treated with Ser/5-HT or fluoxetine. The scheme shows that cultures of U-87 MG cells were settled, 24 hours later treated with fluoxetine (1 to 100 μM) or Ser/5-HT (1 to 1000 μM) for 5 hours, and cell viability was analyzed using MTT. Percentage of viable cells in negative control (culture medium) or positive control (10% DMSO in culture medium) for cell death, and Ser/5-HT- (1 to 1000 μM) or fluoxetine- (1 to 1000 μM) treated cell cultures. Each bar represents the data of an experiment performed in triplicate.
(TIF)

S5 Fig Anti-TNF antibody hampered the rIFNγ-induced augment of U-87 MG cells infection by *T*. *cruzi*. U-87 MG cells were settled, 4 hours later treated with rIFNγ (10 ng/mL) in the absence or presence of the anti-TNF (10 μg/mL) infliximab antibody for 20 hours and infected with cell culture-derived trypomastigote forms using a multiplicity of infection of 5:1 (parasite: cell) and interaction period of 4 hours. The percentage of infected cells and the number of intracellular forms were counted to determine the infection index (Infection Index = percentage of infected cells x number of parasites per infected cell). The graph shows the infection index of an experiment performed in triplicate. The bars show the means ± SD. *, *p* < 0.05, rIFNγ-treated cell culture compared with vehicle-treated cells. ^#^, *p* < 0.05, comparing the presence and the absence of anti-TNF antibodies in rIFNγ-treated cell cultures.
(TIF)

S1 Table Descriptive data of the values used to build graphs. (PDF)
