# Supplementary figures and images for: The beneficial effect of fluoxetine on behavioral and cognitive changes in chronic experimental Chagas disease unveils the role of serotonin fueling astrocyte infection by Trypanosoma cruzi
Source: PLoS Negl Trop Dis. 2024 May 22;18(5):e0012199. doi: 10.1371/journal.pntd.0012199 (PMC11149870; doi:10.1371/journal.pntd.0012199)

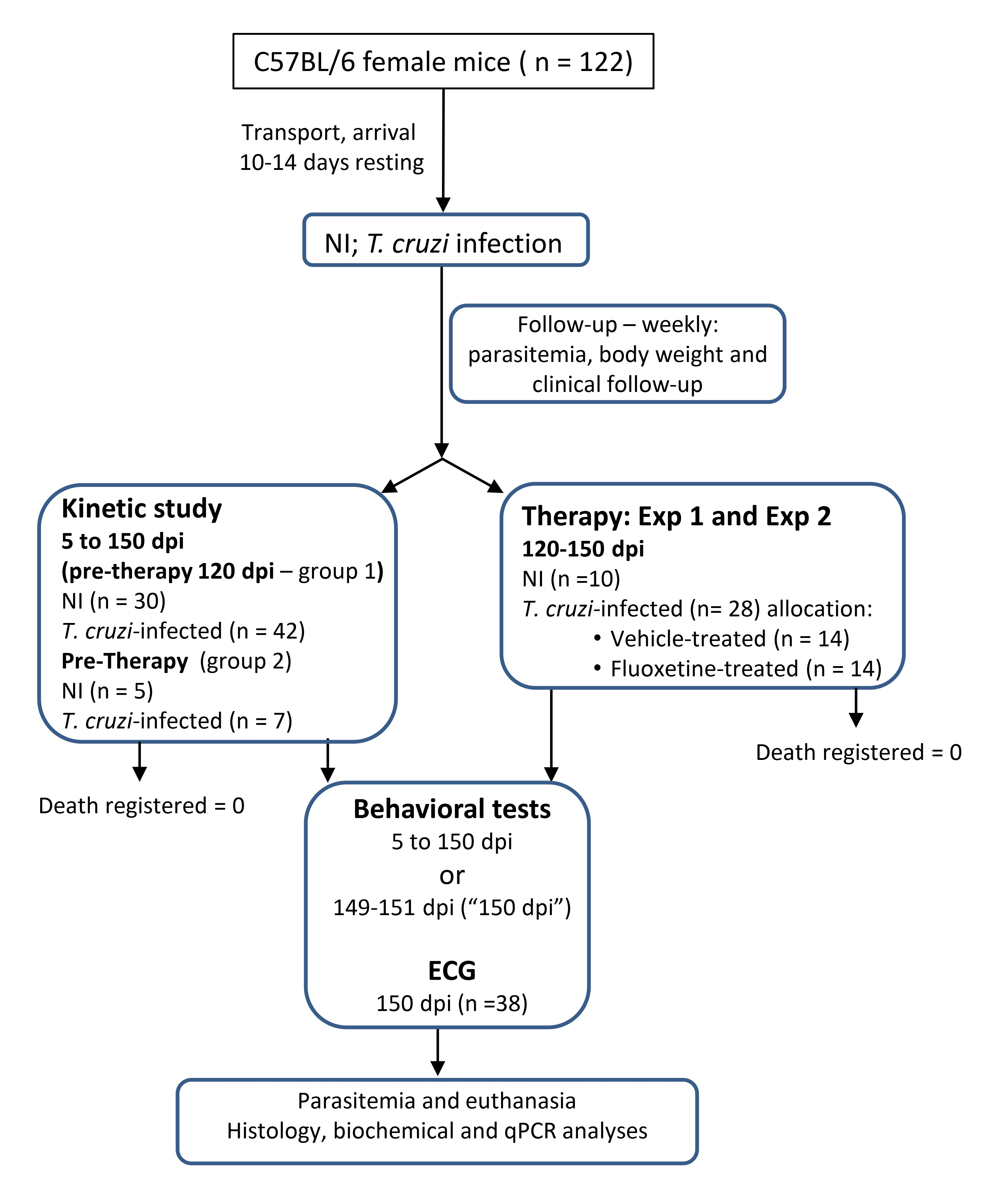

Supplement: S1 Fig — The number of C57BL/6 mice non-infected (NI) and infected with 100 bt forms of the Colombian Type I strain (T. cruzi) used to assess the behavioral and cognitive profiles as a kinetic study (5 to 150 days postinfection, dpi) and to study the effects of vehicle (Veh) and fluoxetine (Fx) administration on the analyzed parameters, including behavioral, and cognitive tests, and electrocardiographic (ECG) registers. Three independent experiments were performed. (TIF) [file pntd.0012199.s001.tif]

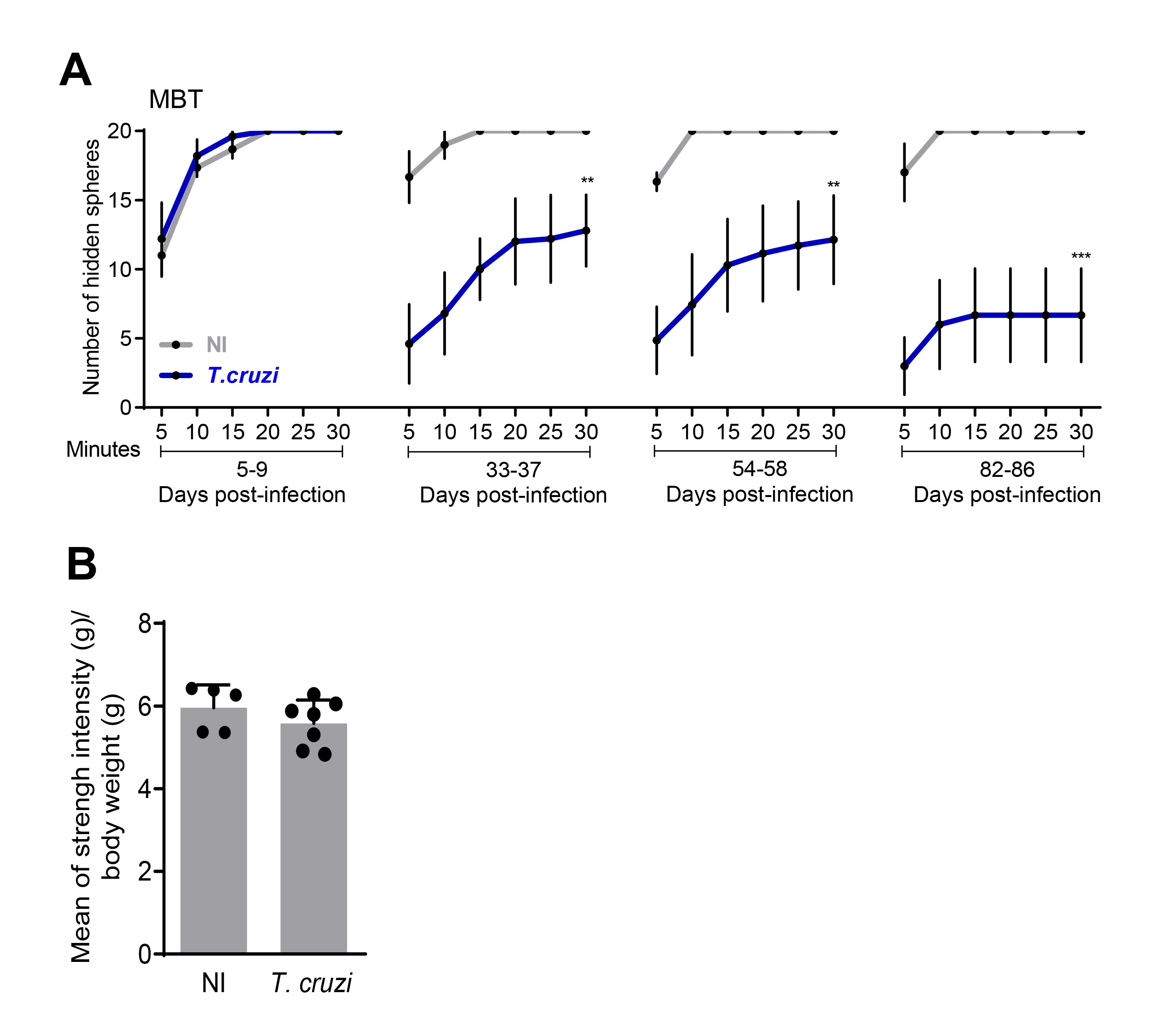

Supplement: S2 Fig — Non-infected controls (NI) and mice infected with 100 bt forms of the Colombian Type I strain (T. cruzi) were submitted to (A) marble burying test (MBT) from 5–9 up to 82–89 days postinfection (dpi), and (B) a non-invasive method to assess the strength of the muscle of mice limbs using grip strength meter test (GMST), at 120 dpi. Color code: (A) Grey lines indicate NI controls, and blue lines T. cruzi-infected mice. (B) Grey bars indicate normal values compared with NI controls. (A) Each dot represents the means of buried marble at the analyzed moment. (B) Each dot represents a mouse. The data are shown as the means ± SD. **, p < 0.01 and ***, p < 0.001, T. cruzi-infected compared with NI controls. (TIF) [file pntd.0012199.s002.tif]

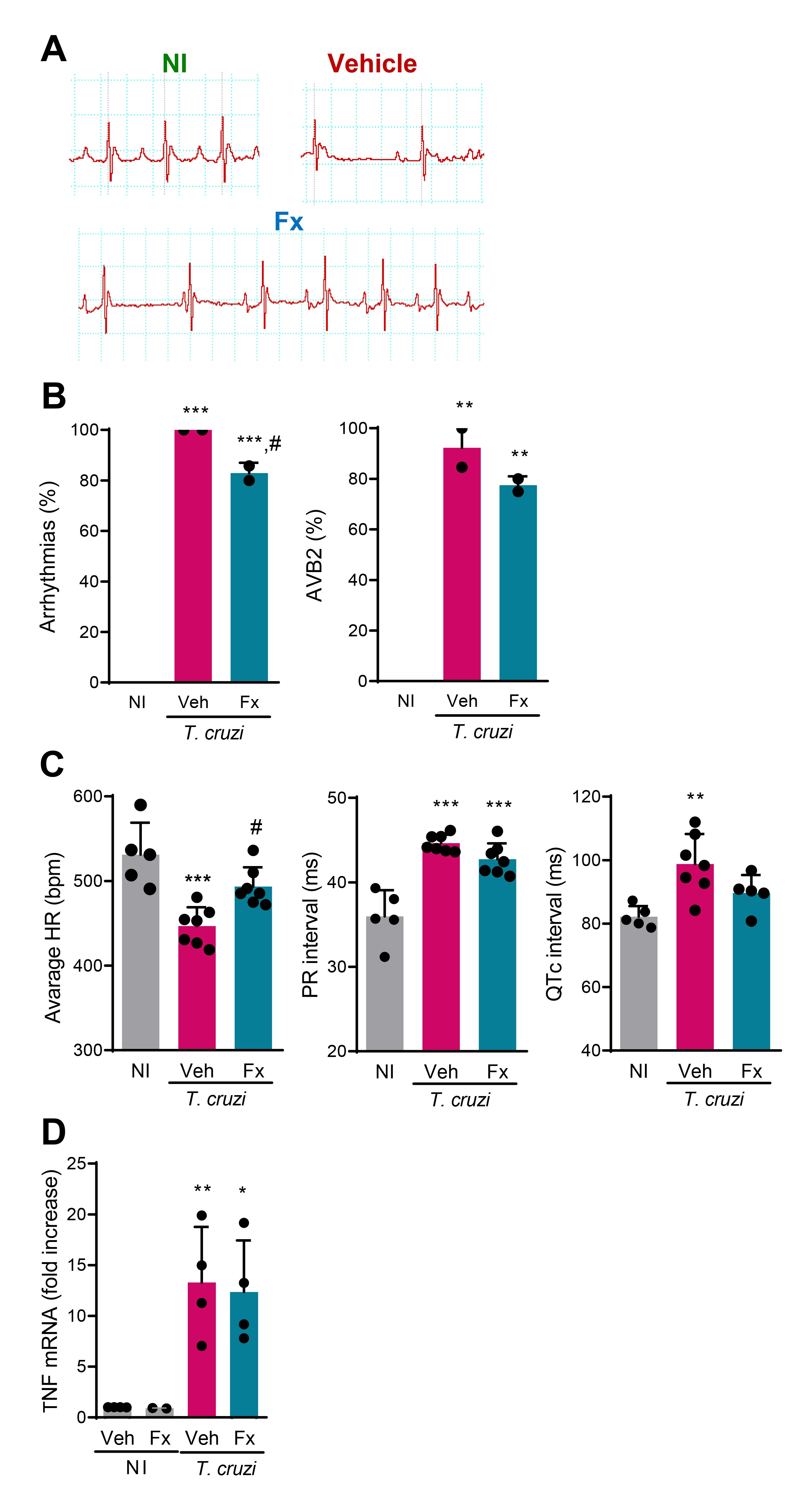

Supplement: S3 Fig — Non-infected controls (NI) and mice infected with 100 bt forms of the Colombian Type I strain (T. cruzi) were submitted to an electrocardiographic (ECG) study and TNF expression in the heart tissue at 150 days postinfection (dpi). (A) Representative ECG register segments of NI and T. cruzi-infected mice treated with vehicle (Veh) or fluoxetine (Fx) from 120–150 dpi. (B) Frequency of mice afflicted by arrhythmias and atrioventricular blocks (AVB2). Each dot represents the percentage of afflicted mice in one independent experiment. (C). Average heart rate (HR; beats per minute, bpm), Group data showing PR interval (ms) and dispersion of the QTc interval (ms). (D) TNF mRNA expression in the heart tissue at 150 dpi. (C-D) Each dot represents a mouse. Results representative of two independent experiments. Color code: Grey bars indicate non-infected controls (NI), pink bars show Veh-treated and blue bars indicate Fx-treated infected mice. The data are shown as the means ± SD. *, p < 0.05, **, p < 0.01 and ***, p < 0.001, T. cruzi-infected compared with NI controls. #, p < 0.05, Fx-treated compared with Veh-treated T. cruzi-infected mice. (TIF) [file pntd.0012199.s003.tif]

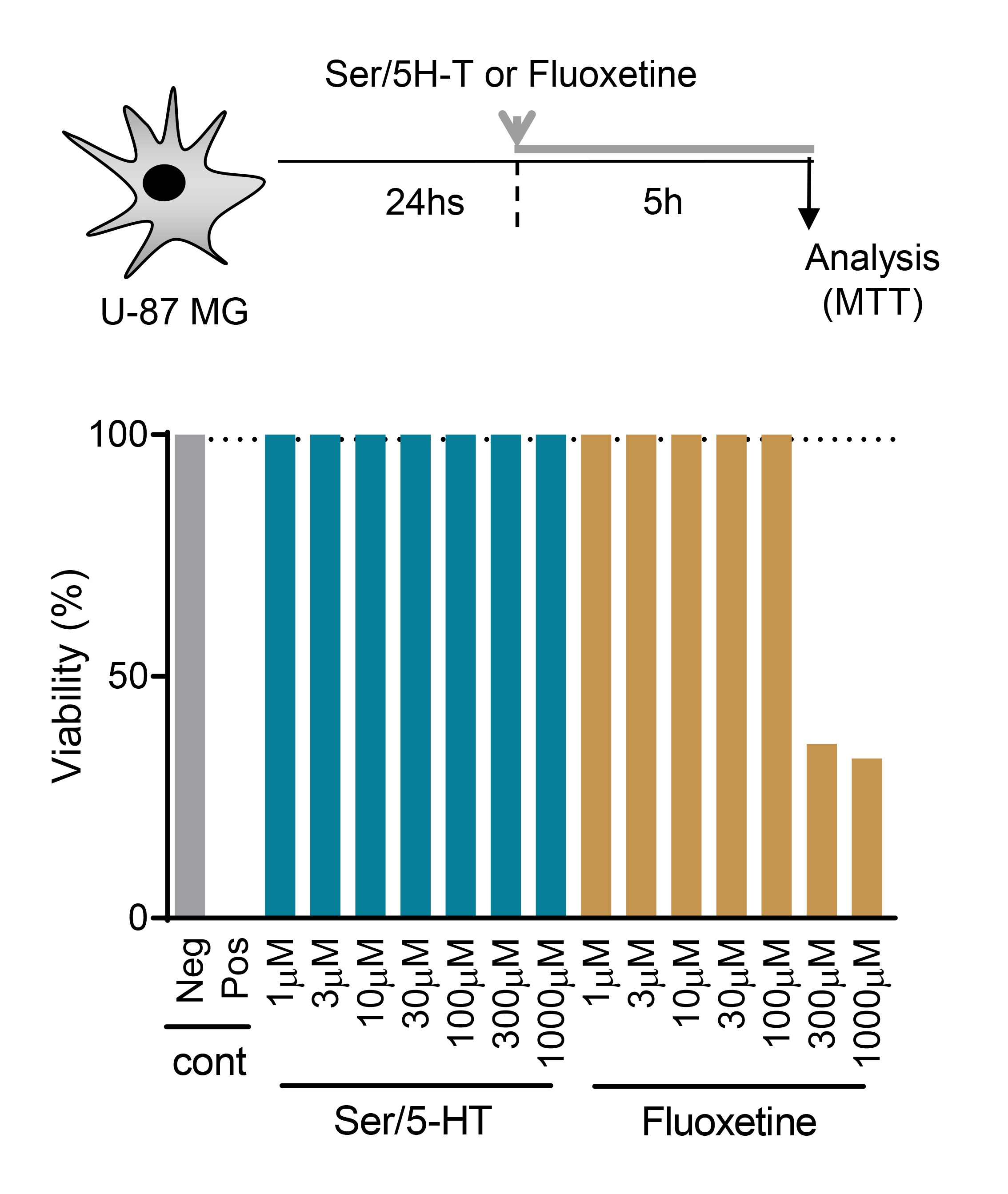

Supplement: S4 Fig — The scheme shows that cultures of U-87 MG cells were settled, 24 hours later treated with fluoxetine (1 to 100 μM) or Ser/5-HT (1 to 1000 μM) for 5 hours, and cell viability was analyzed using MTT. Percentage of viable cells in negative control (culture medium) or positive control (10% DMSO in culture medium) for cell death, and Ser/5-HT- (1 to 1000 μM) or fluoxetine- (1 to 1000 μM) treated cell cultures. Each bar represents the data of an experiment performed in triplicate. (TIF) [file pntd.0012199.s004.tif]

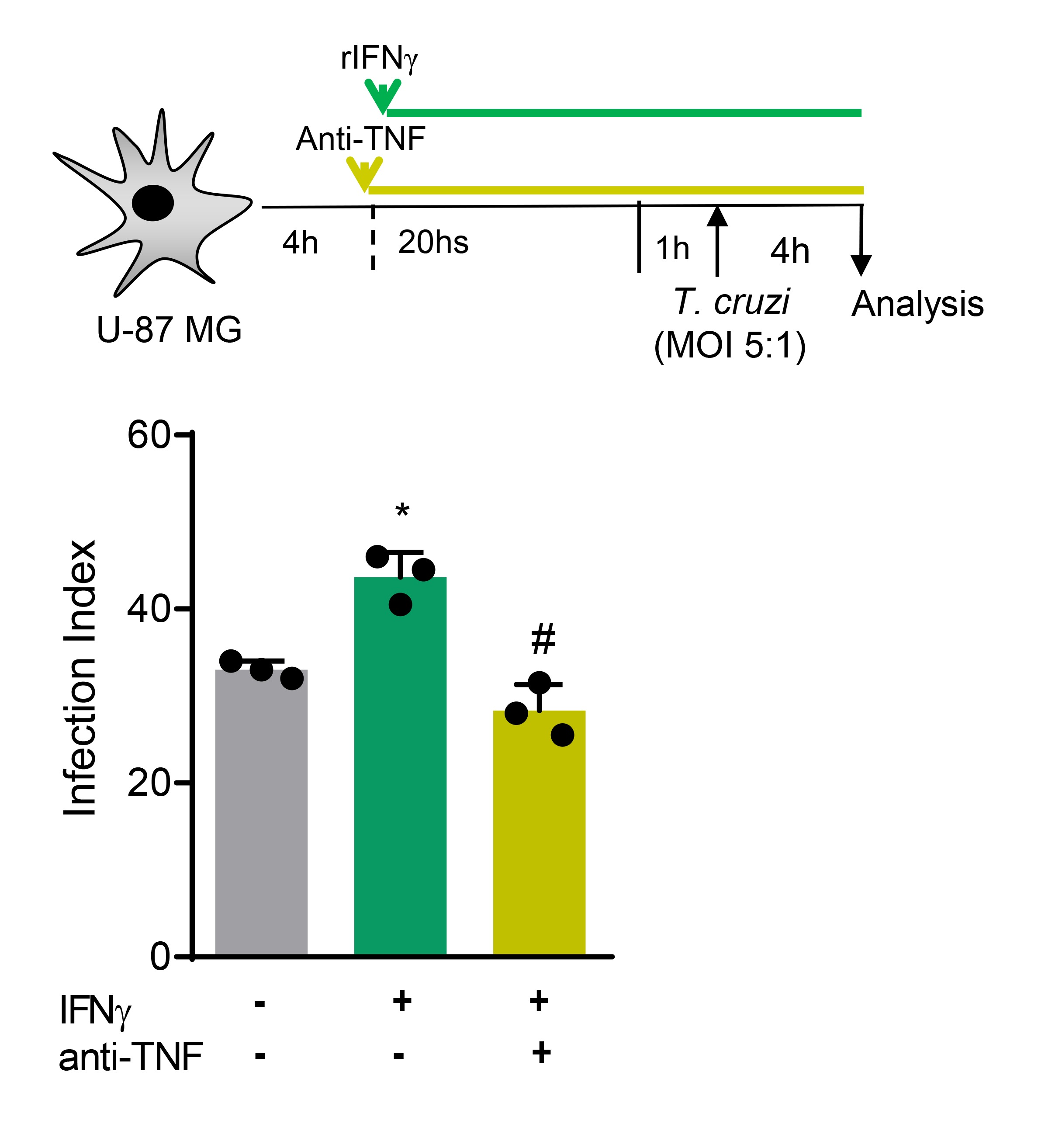

Supplement: S5 Fig — U-87 MG cells were settled, 4 hours later treated with rIFNγ (10 ng/mL) in the absence or presence of the anti-TNF (10 μg/mL) infliximab antibody for 20 hours and infected with cell culture-derived trypomastigote forms using a multiplicity of infection of 5:1 (parasite: cell) and interaction period of 4 hours. The percentage of infected cells and the number of intracellular forms were counted to determine the infection index (Infection Index = percentage of infected cells x number of parasites per infected cell). The graph shows the infection index of an experiment performed in triplicate. The bars show the means ± SD. *, p < 0.05, rIFNγ-treated cell culture compared with vehicle-treated cells. #, p < 0.05, comparing the presence and the absence of anti-TNF antibodies in rIFNγ-treated cell cultures. (TIF) [file pntd.0012199.s005.tif]
