# Supplementary material for: The beneficial effect of fluoxetine on behavioral and cognitive changes in chronic experimental Chagas disease unveils the role of serotonin fueling astrocyte infection by Trypanosoma cruzi
Source: PLoS Negl Trop Dis. 2024 May 22;18(5):e0012199. doi: 10.1371/journal.pntd.0012199 (PMC11149870; doi:10.1371/journal.pntd.0012199)
Supplement: S1 Table — (PDF) [file pntd.0012199.s006.pdf]

Fig 1A

NI						T. cruzi					
0	0	0	0	0	0	0	-0.5	-0.5	-1	-1	-1
0	0	0	0	0	0	0	0	0	-1	-1	-1
0	0	0	0	0	0	0	0	0	-1	-1	-1
0	0	0	0	0	0	0	0	0	1	1	1
0	0	0	0	0	0	0	0	0	1	1	1
0	0	0	0	0	0	0	0	0	0	-1	-1
0	0	0	0	0	0	0	0	0	0	0	-1

Fig 1A Parasitemi parasites/mL

**Day/Mous**	**1**	**2**	**3**	**4**	**5**	**6**	**7**	**8**	**9**	**10**	**11**	**12**	**13**	**14**
**0**	0	0	0	0	0	0	0	0	0	0	0	0	0	0
**7**	0	0	0	0	0	0	0	0	0	0	0	0	0	600
**14**	9333	0	0	0	9333	3111	0	6222	3111	15555	3111	0	0	4300
**21**	37332	6222	12444	0	18666	0	0	37332	0	24888	0	0	12444	13600
**28**	105774	174216	31110	68442	298656	24888	12444	62220	24888	118218	49776	24888	155550	21700
**35**	31110	130662	68442	242658	111996	80886	111996	435540	49776	118218	55998	55998	124440	29800
**42**	105774	105774	55998	391986	31110	199104	155550	329766	298656	149328	62220	62220	87108	4300
**50**	12444	118218	93330	130662	31110	55998	18666	49776	43554	223992	55998	12444	6222	2400
**60**	24888	24888	87108	87108	24888	62220	74664	62220	18666	136884	12444	0	37332	6200
**70**	18666	37332	6222	49776	6222	31110	6222	12444	0	49776	6222	6222	0	0
**120**	0	0	0	6222	0	6222	0	0	0	6222	0	0	6222	1100
**135**	6222	0	0	0	6222	0	0	0	0	0	0	0	0	450
**150**	0	6222	0	0	0	0	0	0	0	0	0	0	0	0

* = NI vs T cruzi Veh

Fig 1B

NI	T.cruzi
93	1140
135	710
18	487
45	593
	537
	1140
	916
**p-Value**	<0.001

Fig 1B

NI						T.cruzi				
20	18	13	13	18		7	2	0	2	0
20	20	20	20	20		11	7	0	7	0
20	20	20	20	20		11	9	0	9	0
20	20	20	20	20		11	9	0	9	0
20	20	20	20	20		11	9	0	9	0
20	20	20	20	20		11	9	0	9	0
**p-Value**	<0.001									

Fig 1C

NI	T.cruzi		NI	T.cruzi
5	2		23.6	12
6	1		39	9
4	2		46.3	11.6
7	1		41.6	8.4
9	1		52	10.5
	1			9.8
**p-Value**	<0.001		**p-Value**	<0.001

Fig 1D

NI	T.cruzi		NI	T.cruzi
35	89		34.02	110.8
37	101.7		40	100
40	81.54		39	96
28	105.41		33.38	111
33	87.38		48	95.4
	102			110.4
	121			
**p-Value**	<0.001		**p-Value**	<0.001

Fig 1E

NI	T.cruzi		NI	T.cruzi
0.496	0.300		0.449	0.406
0.579	0.411		0.508	0.538
0.560	0.429		0.451	0.482
0.382	0.510		0.4	0.680
0.525	0.513			0.334
0.720	0.308			0.409
**p-Value**	<0.05		**p-Value**	>0.05

Fig 1F

	T.cruzi			T.cruzi	
NI	Acute	Chronic	NI	Acute	Chronic
1	1243	2376	1	0.959	0.709
1	375	925	1	0.825	0.885
1	1791	3199	1	0.820	0.753
0.9999	1174	9149	0.999	0.735	0.482
			1	0.539	0.946
**p-Value**	<0.01	<0.01	**p-Value**	>0.05	<0.05
				p = 0.053	

Fig 1G

NI	T.cruzi	NI	T.cruzi
1	1	1	1
1	0.95	1	0.9
1	0.927	1	0.95
0.999	0.852	0.9	0.85
**p-Value**	>0.05	**p-Value**	>0.05

Fig 1H

NI	T.cruzi	NI	T.cruzi
2.4	6.25	11.09	32.53
0.11	8.65	7.54	14.28
0	110.98	3.21	9.69
1.88	305.55	13.67	135.63
0.34	12.89	6.42	66.77
0	8.91	0	85.84
0.36	10.44	3.62	35.91
	8.34		15.21
	16.99		57.44
	132.48		64.05
	8.43		32.32
	24.31		40.5
**p-Value**	<0.001	**p-Value**	<0.01

	* = NI vs T cruzi V	* = NI vs T cruzi F	# = T cruzi Veh vs T cruzi Fx												
	**Fig 2B**														
**Minutes**	**NI**														
**5**	8	12	4	18	15	17	20	8	17	20					
**10**	12	20	12	20	20	20	20	16	20	20					
**15**	20	20	20	20	20	20	20	20	20	20					
**20**	20	20	20	20	20	20	20	20	20	20					
**25**	20	20	20	20	20	20	20	20	20	20					
**30**	20	20	20	20	20	20	20	20	20	20					
**Minutes**	**T.cruzi-Veh**														**p-Value**
**5**	0	0	0	0	4	11	10	4	0	8	0	0	0	9	*<0.001
**10**	0	5	0	10	0	20	20	12	0	8	0	0	0	9	*<0.001
**15**	0	5	0	10	11	20	20	16	0	8	0	0	0	9	*<0.001
**20**	0	5	0	10	11	20	20	16	0	8	0	0	0	9	*<0.001
**25**	0	5	0	10	17	20	20	16	0	8	0	0	0	9	*<0.001
**30**	0	5	0	10	17	20	20	16	0	8	0	0	0	9	*<0.001
**Minutes**	**T.cruzi-Fx**														**p-Value**
**5**	8	12	16	4	4	12	13	20	15	10	13	12	16	4	#<0.001
**10**	20	16	20	13	10	20	20	20	20	10	20	16	20	13	#<0.001
**15**	20	20	20	20	20	20	20	20	20	10	20	20	20	20	#<0.001
**20**	20	20	20	20	20	20	20	20	20	10	20	20	20	20	#<0.001
**25**	20	20	20	20	20	20	20	20	20	10	20	20	20	20	#<0.001
**30**	20	20	20	20	20	20	20	20	20	10	20	20	20	20	#<0.001

Fig 2C

	T.cruzi				T.cruzi	
NI	Veh	Fx		NI	Veh	Fx
8	2	3		54.6	13.3	37
8	1	1		51.6	10	41.6
7	2	3		48.6	11	51
6	2	3		38.3	25	24
8	1	2		47.7	18	29
	1	2			15	
**p-Value**	*<0.001	*<0.001		**p-Value**	*<0.001	*<0.05
		# p = 0.06				# <0.001

Fig 2D

	T.cruzi	
NI	Veh	Fx
202	38	102
213	35	95
290	33	90
287	28	115
207	22	122
	29	129
	30	138
**p-Value**	*<0.001	*<0.001
		#<0.001

Fig 2E

	T.cruzi	
NI	Veh	Fx
35	127	26
27	98	30
38	102	27
35	119	25
34	123	37
	135	15
	118	23
**p-Value**	*<0.001	#<0.001

Fig 2F

	T.cruzi				T.cruzi	
NI	Veh	Fx		NI	Veh	Fx
0.58	0.42	0.63		0.36	0.62	0.28
0.63	0.53	0.72		0.31	0.53	0.35
0.54	0.37	0.66		0.28	0.47	0.3
0.56	0.40	0.68		0.35	0.72	0.56
0.68	0.29	0.53		0.29	0.52	0.40
	0.24	0.58			0.43	0.29
	0.31	0.62			0.51	0.25
p-Value	* <0.001	# <0.001		p-Value	* <0.01	# <0.01

Fig 3B

	T.cruzi				T.cruzi	
NI	Veh	Fx		NI	Veh	Fx
0.22	2.22	0.7		0.3	0.38	0.24
0.31	1.91	0.85		0.2	0.24	0.18
0.53	1.86	0.81		0.26	0.28	0.29
p-Value	* <0.001	* <0.05		p-Value	* >0.05	* >0.05
		# <0.001				# >0.05

Fig 3D

	T.cruzi				T.cruzi	
NI	Veh	Fx		NI	Veh	Fx
0.5	1.5	0.73		0.13	0.92	0.22
1	1.62	1.11		0.07	0.41	0.19
0.37	1.52	1.07		0.28	0.54	0.21
p-Value	* <0.01	* >0.05		p-Value	* <0.05	*>0.05
		# <0.05				#<0.05

Fig 4A

	T.cruzi				T.cruzi	
NI	Veh	Fx		NI	Veh	Fx
0.190	0.473	0.355		0.253	0.536	0.222
0.243	0.463	0.243		0.255	0.596	0.362
0.258	0.399	0.358		0.299	0.443	0.294
**p-Value**	* <0.01	* >0.05		**p-Value**	* <0.01	* >0.05
		# <0.05				# <0.01

Fig 4B

	T.cruzi				T.cruzi	
NI	Veh	Fx		NI	Veh	Fx
1	0.51	2.441		1	0.323	1.26
0.999	0.442	2.597		1	0.639	0.449
0.998	0.436	3.289		0.98	0.314	0.642
	0.82	0.99				
**p-Value**	* <0.05	* <0.05		**p-Value**	* <0.01	* >0.05
		# <0.01				# >0.05

Fig 4C

	T.cruzi	
NI	Veh	Fx
114	115	130
120	119	108
118	116	111
**p-Value**	* >0.05	* >0.05
		# >0.05

4C insert

**BDNF**	**OD_{450}**
250	0.662
125	0.298
62.5	0.163
31.25	0.074
15.62	0.028
7.812	0.006
0	0.000

Fig 5B					
	T.cruzi			T.cruzi	
NI	Veh	Fx	NI	Veh	Fx
0	1.623	0.0018	0.0001	0.811	0.0012
0	2.057	0.0002	0.0003	0.933	0.001
5,3E-05	1.722	0.0003	0.0000	0.728	0.000121
	* <0.05	# <0.05		* <0.001	# <0.001

Fig 6B				Ser			
NT	Fluox 20		Veh	**0.01**	**0.1**	**1**	**10**
11.2	10		9.6	10	11.4	16.2	21.8
11.8	10.6		9.8	10.4	10	15.4	21
**p-Value**	>0.05			>0.05	>0.05	<0.01	<0.001

* = NT vs Ser | # = Ser vs Ser+ Fx

Fig 6C					
NT	Fluox 20	Ser 1	Ser 10	Fx 20 + S	Fx 20 + Ser 10
11.2	10	16.6	21.6	12.2	12.8
11.5	10.2	17.2	20.8	14	12.8
10.6	10	16.4	22.2	11.6	12.6
**p-Value**	>0.05	*<0.001	*<0.001	#<0.001	#<0.001

* = NT vs Ser or IFN | # = Ser or IFN vs Ser+ IFN

Fig 7			
NT	IFN	Ser	IFN+Ser
9.6	11.8	16.2	18.2
9.8	11	15.4	18.6
**p-Value**	* <0.05	*<0.001	*<0.001
	#<0.01	#<0.05	

* = NT vs Ser or T | # = Ser or TNF vs | & = Ser or Ser+ TNF vs Ser+ TNF + Fx

Fig 8B					
NT	TNF	Fluox 20	Ser 1	TNF + S	TNF + FX + Ser1
11.2	16.2	10.2	16.4	19.6	12
11.8	16	10.6	18	19.2	12
10.6	16	10	16		11.8
**p-Value**	*<0.001	*>0.05	*<0.001	*<0.001	*>0.05
	#<0.001		#<0.01		
			&<0.001	&<0.01	

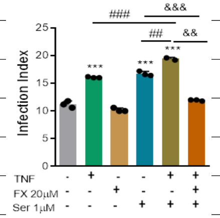

Fig8B

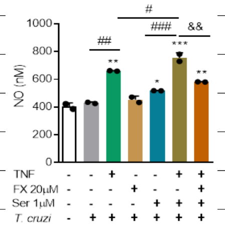

Fig8C

* = NI vs All | # indicatec conditions | & indicated conditions

Fig 8C	*T. cruzi*					
**NI**	NT	TNF	Fluox 20	Ser 1	TNF + S	TNF + FX + Ser1
387.4	435.6	662.9	434	519	729.7	583.1
423	426.3	659.8	472.1	517.6	781	581.2
**p-Value**	*>0.05	*<0.01	*>0.05	*<0.05	*<0.001	*<0.01
		#<0.01		#<0.001	#<0.01	
					&<0.01	

* = NI vs All | # indicatec conditions | & indicated conditions

Fig 8D	*T. cruzi*					
**NI**	NT	TNF	Fluox 20	Ser 1	TNF + S	TNF + FX + Ser1
9.775	10.471	10.920	9.855	12.169	12.151	10.512
9.962	10.064	10.504	10.145	12.197	12.847	10.498
9.912	10.549	10.871	9.715	11.283	13.149	10.050
**p-Value**	*>0.05	*>0.05	*>0.05	*<0.001	*<0.001	*>0.05
		#<0.01		#<0.05		
				&<0.01	&<0.001	

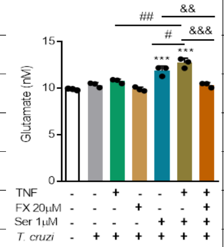

Fig8D

S2A Fig

	NI		T. cruzi	
**5.9**	Mean	SD	Mean	SD
**5**	11	2.2	12.2	5.8
**10**	17.3	0.94	18.2	2.7
**15**	18.7	0.94	19,6	0.9
**20**	20	0	20	0
**25**	20	0	20	0
**30**	20	0	20	0
**37-39**				
**5**	16.7	2.6	4.6	6.4
**10**	19	1.4	6.8	6.6
**15**	20	0	10	4.9
**20**	20	0	12	7
**25**	20	0	12.2	7.1
**30**	20	0	12.8	5.8
**54-58**				
**5**	16.3	0.94	4.8	6.4
**10**	20	0	7.4	9.6
**15**	20	0	10.3	8.8
**20**	20	0	11.1	9.1
**25**	20	0	11.7	8.4
**30**	20	0	12.1	8.4
**82-89**				
**5**	17	2.9	3	3.6
**10**	20	0	6	5.6
**15**	20	0	6.7	5.9
**20**	20	0	6.7	5.9
**25**	20	0	6.7	5.9
**30**	20	0	6.7	5.9

S2B Fig

NI	T.cruzi
5.37	5.87
6.27	4.91
6.42	5.31
5.36	4.83
6.38	6.05
	5.8
	6.27
**p-Value**	*>0.05

S3B Fig

**% ART**			**% AVB2**		
	T.cruzi			T.cruzi	
NI	Veh	Fx	NI	Veh	Fx
0	100	80	0	85	80
0	100	86	0	100	75
**p-Value**	* <0.001	* <0.001	**p-Value**	* <0.01	* <0.01
		# <0.05			# >0.05

S3C Fig

**Average HR**			**PR interval**			**QTc interval**		
	T.cruzi			T.cruzi			T.cruzi	
NI	Veh	Fx	NI	Veh	Fx	NI	Veh	Fx
490.6	430.7	536.3	35.71	43.99	43.41	80.09	92.73	90.86
507	463.1	485.5	39.33	45.42	46.03	83.74	94.49	90.3
536.6	453	491.1	31.18	44.14	41.39	78.73	101.6	89.6
589.9	418.9	485.2	35.57	45.38	40.73	81.17	108.2	80.81
531.3	454.6	474.9	38.03	43.61	41.25	87.24	112	96.7
	480.8	471.9		43.75	43.94		84.18	90.3
	426.7	510		46.15	42.44		98.33	89.6
**p-Value**	* <0.001	* >0.05	**p-Value**	* <0.001	* <0.001	**p-Value**	* <0.01	* >0.05
		#<0.05			# >0.05			# >0.05

S3D Fig

TNF mRNA

**NI**			**T.cruzi**	
Veh	Fx		Veh	Fx
1	0.93		7.05	7.80
1	0.89		14.97	13.26
1			11.23	9.18
1			19.89	19.15
**p-Value**			* <0.01	* <0.05
				# >0.05

S4 Fig **Viability (%)**

Neg	Pos	**Ser/5H-T**						
100	0	1µM	3µM	10µM	30µM	100µM	300µM	1000µM
		100	100	100	100	100	100	100

Fx

1µM	3µM	10µM	30µM	100µM	300µM	1000µM
100	100	100	100	100	36	33

S5 Fig

NT	IFN	IFN+anti-TNF
32	46	31.5
33	44.5	28
34	40.5	25.5
**p-Value**	* <0.01	* >0.05
		# <0.05
